# Bioactivity studies of organometallic Cp*Rh(iii) and Au(i) NHC complexes bioconjugated to integrin binding peptides

**DOI:** 10.1039/d6md00713a

**Published:** 2026-08-31

**Authors:** Felix A. Böhm, Miriam Caviglia, Bernd Schulz, Claudia Schmidt, Roland A. Fischer, Richard H. Fish, Iogann Tolbatov, Alessandro Marrone, Guillermo Moreno-Alcántar, Angela Casini

**Affiliations:** a Chair of Medicinal and Bioinorganic Chemistry, Department of Chemistry, School of Natural Sciences, Technical University of Munich Garching Germany angela.casini@tum.de; b Chair of Inorganic and Metal-Organic Chemistry, Department of Chemistry, School of Natural Sciences, Technical University of Munich Garching Germany; c School of Science and Technology, Chemistry Division (ChIP), University of Camerino Camerino Italy; d Lawrence Berkeley National Laboratory, University of California Berkeley California USA; e Department of Chemical, Physical, Mathematical and Natural Sciences, University of Sassari Via Vienna 2 07100 Sassari Italy; f Department of Pharmacy, University “G. D'Annunzio” of Chieti-Pescara Via dei Vestini 31 66100 Chieti Italy amarrone@unich.it; g Center for Cooperative Research in Biomaterials (CIC biomaGUNE), Basque Research and Technology Alliance (BRTA) Paseo de Miramon 194 20014 Donostia-San Sebastián Spain gmorenoalcantar@cicbiomagune.es

## Abstract

Integrins are pivotal targets in cancer therapy development due to their overexpression in many tumor cells and neovascular tissue. Arginine–glycine–aspartic acid (RGD) sequence-containing peptides are established ligands for targeting integrins; approximately half of all known integrins recognize this sequence, enabling selective delivery of drugs and imaging agents to tumors and their vasculature. Despite the widespread application of cyclic RGD (cRGD) peptides in oncological research, the number of reported RGD conjugates featuring organometallic payloads remains limited. In this work, we present new Au(i) and Rh(iii) organometallic cRGD conjugates and study their bioactivity by a combination of experimental and theoretical methods. The three organometallic bioconjugates, labelled **Rh-RGD**, **Au-RGD** and **Au-RGD**_**2**_, were obtained by integrating Cp*Rh(iii) and Au(i) N-heterocyclic carbene (NHC) complexes into the cyclic peptide c(RGDyK) through chemoselective Rh(iii) coordination at tyrosine and Au(i) NHC wingtip conjugation at the lysine amino group, enabling a direct comparison of distinct organometal–peptide conjugation strategies. Tight-binding density functional theory (DFT) calculations at the αvβ3 Cilengitide pocket indicated that the **Rh-RGD** complex maintained the peptide recognition mode and dissociation energetics essentially unchanged, whereas **Au-RGD** engaged weakly with the binding pocket. The *in vitro* antiproliferative activity studies have shown that RGD conjugation does not necessarily improve the cytotoxicity of the organometallic complexes. However, inductively coupled plasma mass spectrometry (ICP-MS) revealed clear differences in metal uptake, with monomeric **Au-RGD** efficiently internalized, **Rh-RGD** closely matching its metal precursor, and **Au-RGD**_**2**_ showing negligible accumulation. Altogether, these data pointed to a finely balanced interplay between integrin recognition, steric accessibility, solvation, and metal-centred reactivity, while emphasizing that successful c(RGD)-guided metallopeptides require optimization of organometallic chemistry, and vector design towards the development of the next-generation targeted metallodrugs.

## Abstract 2

Au(i) and Rh(iii) cRGD peptide-conjugates were developed for cancer targeting. Experiments and DFT models showed that metal integration uniquely impacts integrin binding and cellular uptake, guiding future targeted metallodrug design.

## Graphical abstract

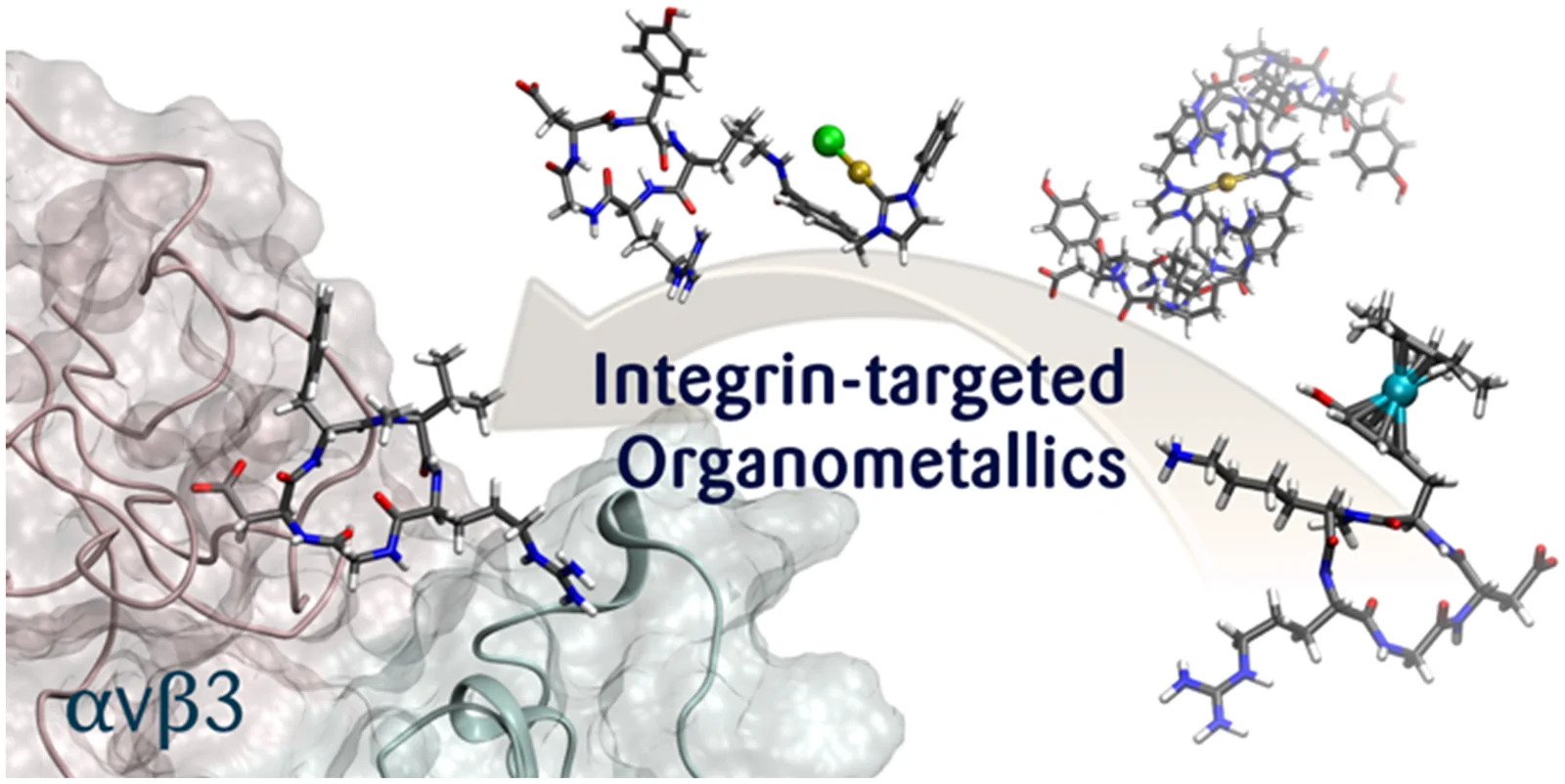

## Introduction

Integrating organometallic fragments, containing at least one carbon–metal bond, into peptides to create a hybrid class of targeted therapeutics known as organometallic peptidomimetics or metallopeptides, has become a powerful strategy in modern oncology.^1^ This approach directly addresses the primary limitations of traditional platinum-based chemotherapies, which include a lack of tumor selectivity, severe systemic side effects, and rapidly emerging drug resistance.^2^ By coupling organometallic complexes of rhodium, ruthenium, iridium, osmium, gold, and iron to specific peptide sequences, researchers have successfully transformed non-selective, highly toxic metal compounds into precise, tumor-homing weapons.^1^

**Fig. 1:**
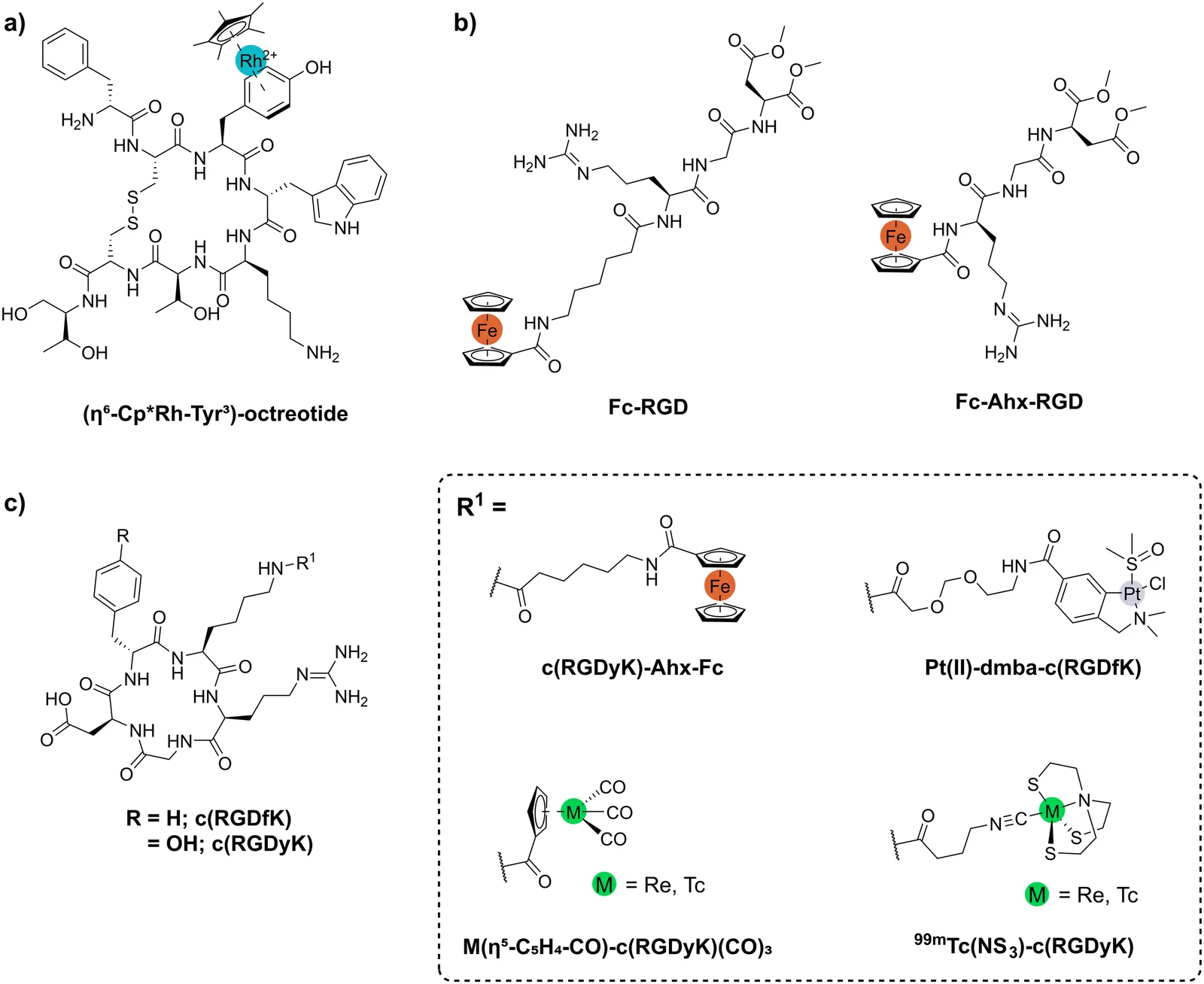
Representative examples of bioorganometallic compounds developed as anticancer agents. a) Structure of [(η^6^-Cp*Rh-Tyr^3^)-octreotide]^2+^. b) Linear RGD-ferrocene (Fc) complexes targeting integrin receptors. c) cRGD derivatives of various metals, including Rh, Fe, Pt, Re, and Tc, for medicinal applications.

Organometallic bioconjugates have been classified into two groups, based on the nature of the organometallic–peptide bond. In most cases, the peptides were coordinated to the metal center of organometallic moieties *via* heteroatoms located either in the peptide backbone or in the amino acid side chains, and were available after insertion of an extra coordination site in metal-bound peptides. Therefore, the bioorganometallic nature arises from M–C bonds between the metal ion and spectator ligands (*e.g.*, CO, N-heterocyclic carbene (NHC), cyclometalated or arene ligands).^3–6^ For example, NHC–Au(i) complexes were conjugated to cysteine (NHC–Au(i)–Cys) and a Leu-Cys (NHC–Au(i)–LeuCys) dipeptide, *via* direct Au–S coordination to the thiol group, yielding amino acid and dipeptide gold bioconjugates.^7^ Alternatively, peptides bearing organometallic ligands could be directly carbon-linked to the metal center, forming i) metal-arenes (metallocenes and “half-sandwich” compounds), ii) carbenes, or iii) cyclometallated compounds.^3,8^ N-heterocyclic carbene complexes, widely used in organometallic catalysis and medicinal chemistry for their high stability and activity, were well-suited for the synthesis of transition-metal peptide conjugates.^8^ In this instance, the wingtip groups of the NHC scaffolds have often been used to introduce amino acid functionalities. However, backbone functionalization of NHCs with peptides has also been reported.^9–11^ Concerning arenes, all the natural amino acids that contain an aromatic side-chain functionality (phenylalanine, tyrosine, and tryptophan) have been metalated with organometallic reagents,^3,4^ forming η^6^ complexes by π-coordination of the metal ion to the aromatic ring. Thus, strategies such as arene-selective derivatization of amino acids, simple peptides, and larger peptides and proteins have been extensively reviewed.^3,4^ A remarkable example was the chemoselective bioconjugation of tyrosine residues in G-protein-coupled receptor (GPCR) peptides, which Fish *et al.* reported to occur in water at room temperature using the air- and water-stable organometallic aqua complex [Cp*Rh(H_2_O)_3_](OTf)_2_.^12^ This reaction proceeds with high chemoselectivity for the tyrosine phenol group, leaving competing functionalities, including amino, carboxyl, disulfide, phenyl, and indole groups unreacted. Three GPCR peptides were conjugated in this manner: [Tyr^1^]-leu-enkephalin, [Tyr^4^]-neurotensin(8-13), and [Tyr^3^]-octreotide ((η^6^-Cp*Rh-Tyr^3^)-octreotide; Fig. 1). The antiproliferative effects of the conjugates were investigated in human breast adenocarcinoma (MCF7) and colon carcinoma (HT29) cell lines *in vitro*. The results showed that the EC_50_ values of the Cp*Rh conjugates were similar to those of the free peptides, indicating that conjugation did not alter the antiproliferative activity of either GPCR peptide.^12^

Among the more widely used peptides, the arginine–glycine–aspartic acid (RGD) sequence is a key element in integrin-targeted therapeutic strategies, enabling specific drug delivery, imaging, and antiangiogenic therapy.^13,14^ Moreover, cyclic RGD peptides (cRGD) have emerged as state-of-the-art integrin-targeted motifs, due to their enhanced conformational constraints, which lead to improved receptor-binding affinity and resistance to proteolytic degradation compared with linear variants. Pioneering work by Kessler and coworkers introduced the cyclic pentapeptide c(RGDf-N(Me)-V), which was later branded as cilengitide.^15,16^

In addition to cyclization, the incorporation of d-phenylalanine and *N*-methylvaline further improved its pharmacological properties, by increasing its resistance to proteolytic degradation. Cilengitide was shown to be an excellent targeting motif for the integrins αvβ3, αvβ5, and α5β1.^15,17^ The capacity of cilengitide to trigger apoptosis in proliferating endothelial cells, suppress cell migration, and interfere with focal adhesion signaling, yielded promising preclinical results, ultimately leading to its advancement into clinical trials for glioblastoma and other solid tumors. Although subsequent phase III trials failed to meet expectations, cilengitide's strong preclinical performance provided the conceptual groundwork for its repurposing as a scaffold for targeted drug-delivery systems.^18,19^ However, cilengitide lacks conjugation handles, severely limiting its therapeutic potential. The closely related cyclic RGD sequences c(RGDfK) and c(RGDyK) overcome this limitation by replacing the valine residues with a lysine, which can be used for common bioconjugation methods on the amino-bearing side chain.^20^ While this change retained a comparable affinity for the αvβ3 integrin, enabling receptor-mediated endocytosis upon binding, the affinities for αvβ5 and α5β1 were reduced by one or two orders of magnitude yet remained in the low micromolar range.^21^ Furthermore, despite the RGD moiety being widely used in medicinal chemistry and biomedical imaging,^22^ including radiopharmaceutical application^23,24^ and bioconjugation to bioactive coordination complexes,^5,25–27^ only a handful of organometallic RGD conjugates (OM-RGDs) have been reported.

Starting with ferrocene (Fc), which has been shown to be the most common metallocene, linear Fc–RGD and Fc–Ahx–RGD (Ahx = 6-aminohexanoic acid spacer) conjugates were synthesized *via* standard amide coupling of peptides with ferrocenylalanine^28^ (Fig. 1), that targeted αvβ3/αvβ5-overexpressing melanoma cells, *via* integrin-mediated cell uptake.^29^ The insertion of the Ahx-type spacer increased cellular uptake in B16 murine melanoma cells up to 83%, compared to 42% for the spacer-free Fc–RGD.^29^ The most potent conjugate, Fc–Ahx–RGD, displayed an EC_50_ of 5.2 ± 1.4 μM toward B16 cells, approximately 9-fold lower than that of free Fc–COOH (EC_50_ = 49.0 ± 5.8 μM), along with a 5-fold higher apoptosis rate, demonstrating that integrin targeting *via* RGD, combined with a flexible spacer, significantly amplifies the cytotoxic efficacy of Fc.^29^ More recently, a similar strategy was used to conjugate Fc to a cyclic integrin-binding peptide (c(RGDyK)), *via* standard amide coupling (c(RGDyK)–Ahx–Fc; Fig. 1), thereby enabling integrin-mediated delivery of the ROS-generating Fc moiety into αvβ3-overexpressing tumor cells.^30^ The conjugate retained low nanomolar αvβ3-affinity, and displayed up to 30-fold greater cytotoxicity than free Fc–COOH in M21 melanoma cells (EC_50_ conjugate c(RGDyK)–Ahx–Fc = 31 μM *vs.* EC_50_ Fc–COOH > 1000 μM), with selective ROS production and DNA damage confirmed exclusively in αvβ3-positive cell lines.^30^

A cyclometalated Pt(ii) complex bearing a carboxylic acid-functionalized C^N-chelating *N*,*N*-dimethylbenzylamine (dmba) ligand was conjugated to c(RGDfK) *via* a PEG spacer, to yield a dual antitumor/antiangiogenic bioconjugate (Pt(ii)–dmba–c(RGDfK); Fig. 1).^31^ In addition to therapeutic applications, organometallic conjugation of radiopharmaceuticals to c(RGDyK) for single-photon emission computed tomography (SPECT) imaging has been explored. Thus, ^99m^Tc and Re complexes of the half-sandwich type [M(η^5^-C_5_H_4_-CO)-c(RGDyK)(CO)_3_] were prepared by conjugation of the cyclopentadienyl–tricarbonyl moiety to the lysine side chain of c(RGDyK), with ^99m^Tc labeling achieved in 49% yield (Fig. 1).^32^ In other studies, the lysine side chain of the peptide was functionalized with a 4-isocyanobutanoic acid linker, to generate a monodentate isocyanide-modified peptide (^99m^Tc(NS_3_)-c(RGDyK); Fig. 1).^33^

**Scheme 1:**
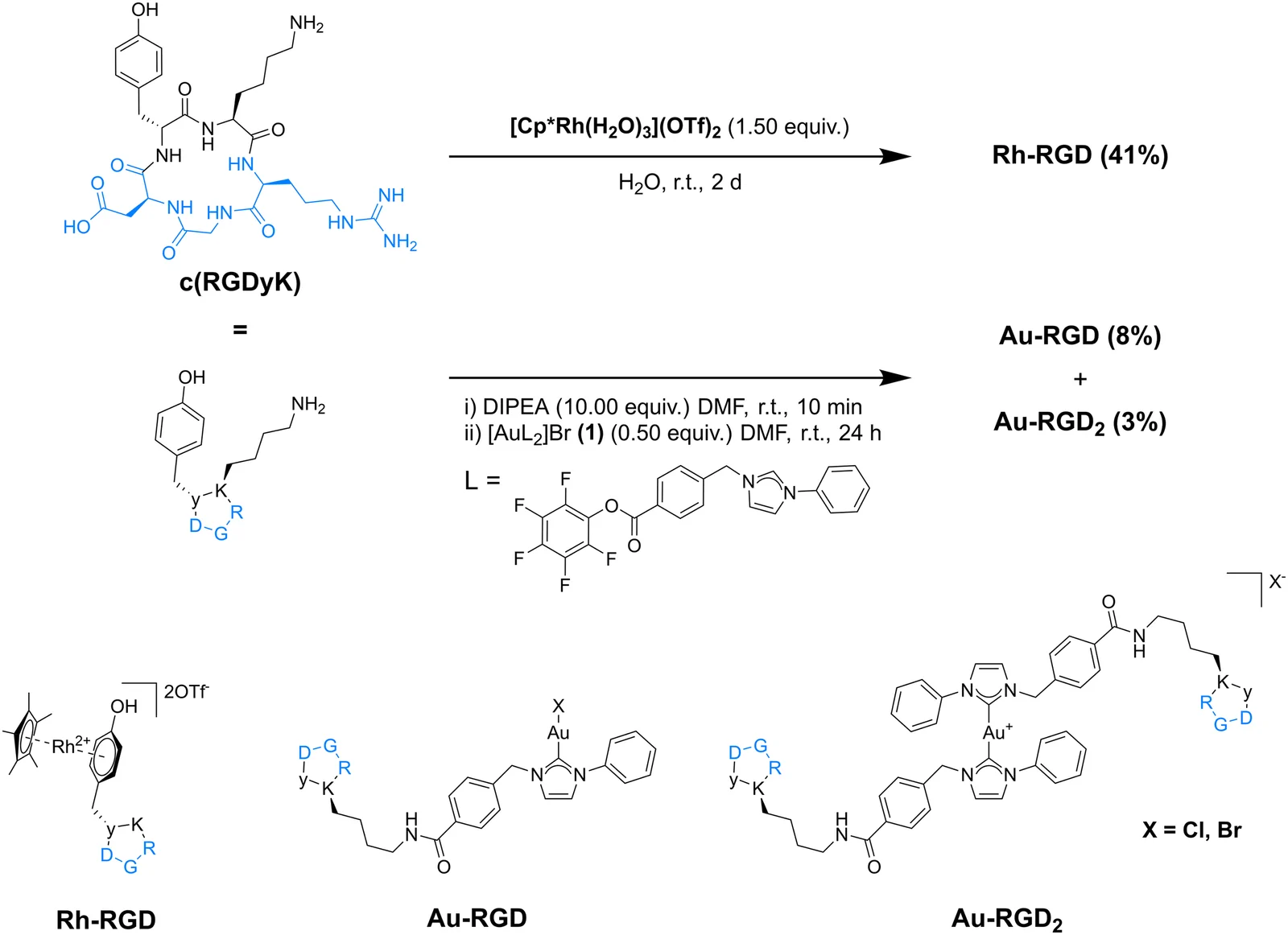
Synthesis of the bioorganometallic complexes **Rh-RGD**, **Au-RGD**, and **Au-RGD**_**2**_ reported in this work.

Given the relevance of RGD peptides in the development of targeted therapeutics and the scarcity of OM-RGD conjugates, this work introduces the first examples of Au(i) and Rh(iii) derivatives within this compound class (Scheme 1). The bioconjugates feature the c(RGDyK) peptide, where the *ε*-amino group of the lysine residue was utilized as the anchor for the introduction of a Au(i)-NHC moiety, while the tyrosine residue, absent in the more commonly employed c(RGDfK) scaffold, serves as a chemoselective organometallic bonding point for direct coordination of an η^6^-Cp*Rh^2+^ moiety.^12^ The obtained metal conjugates have been studied for their integrin receptor-binding affinity, using density functional theory (DFT), and their antiproliferative affinity has been evaluated in a small panel of human cancer cells, with varying levels of integrin expression, *in vitro*. Moreover, the cellular uptake of the conjugates in cancer cells has been studied by inductively coupled plasma mass spectrometry (ICP-MS).

## Results and discussion

The peptide c(RGDyK) was synthesized using a modified procedure from Han *et al.*^34^ The Cp*Rh aqua complex, [Cp*Rh(H_2_O)_3_](OTf)_2_, was synthesized from [Cp*RhCl_2_]_2_ by halide abstraction from the dimeric chlorido precursor [Cp*RhCl_2_]_2_ using silver triflate (AgOTf) in DCM, following the synthetic protocol reported by Fish *et al.*^35^ Employing the intermediates perfluorophenyl 4-(bromomethyl)benzoate (a) and 3-(4-((perfluorophenoxy)carbonyl)benzyl)-1-phenyl-1*H*-imidazol-3-ium bromide (b), the phenyl-substituted Au(i)-bisNHC precursor (1, Scheme 1) was obtained. Further synthetic details for c(RGDyK), [Cp*Rh(H_2_O)_3_](OTf)_2_, (a), (b), and (1) are given in the Experimental section.

As depicted in Scheme 1, the three OM-RGD conjugates named **Rh-RGD**, **Au-RGD**, and **Au-RGD**_**2**_ were obtained *via* the conjugation of the respective OM-precursors to the two peptide binding sites presented by tyrosine and lysine. Interestingly, starting from a Au(i)-bis-NHC precursor (1), the reaction yielded the mono-NHC conjugate **Au-RGD** as a side product, replacing the second NHC handle with a halide (X = Cl and Br). All three compounds could be isolated and characterized after HPLC purification.

**Fig. 2:**
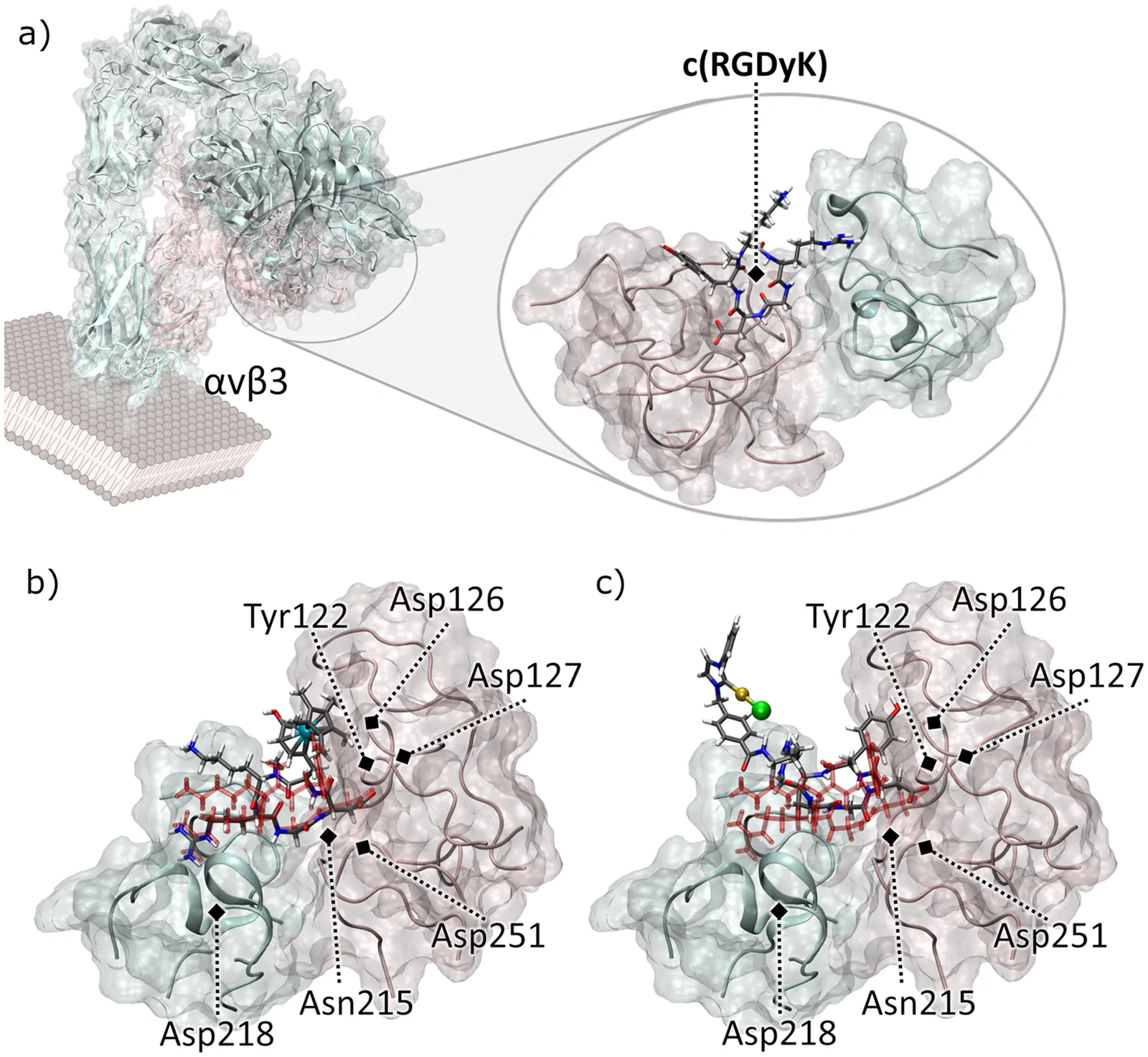
a) Schematic representation of the extracellular fragment of the αvβ3 integrin (PDB 1L5G) depicting the position of the model binding pocket used in the TB-DFT studies, and the inset shows the calculated position of **c(RGDyK)** within the model system. b) and c) Binding modes of **Rh-RGD** and **Au-RGD** complexes to the αvβ3 pocket, as compared with that of **c(RGDyK)** (red trace). The αv and β3 models are rendered as teal and pale red ribbons, respectively; the shown solvent accessible surfaces are modeled with a probe *r* = 1.4 Å.

Insights into the binding of the OM-RGD compounds to integrin were preliminarily gained computationally by investigating the interaction of the peptide conjugates with the αvβ3 integrin at the tight-binding density functional theory (DFT) level of theory using xTB 6.7.1 software,^36–39^ and implicit solvation (water). The reported cilengitide–αvβ3 crystal structure (PDB: 1L5G) was used to generate the binding pocket model (see the Computational methods section for details).^40^ Within this framework, the calculated binding conformation of cilengitide was reproduced satisfactorily, including the key hydrogen-bonding interactions between the arginine side chain and Asp218 of the αv subunit, and between the ligand aspartate and Tyr122/Asn215 of the β3 subunit, supporting the use of this model for comparison of the modified conjugates. The binding conformations of c(RGDyK) and its Cp*Rh-conjugate, **Rh-RGD**, resembled the cilengitide recognition mode, by forming essentially the same hydrogen bond patterns (Fig. 2). Interestingly, the [η^6^-Cp*Rh-Tyr]^2+^ complex of **Rh-RGD** was situated close to a negatively charged region, which was formed by Asp126, Asp127, and Asp251, by concomitantly enhancing the target–peptide interaction energy. Indeed, the calculated αvβ3 affinities of cilengitide, c(RGDyK), and **Rh-RGD**, estimated from the target–ligand dissociation energies of 119, 107, and 105 kcal mol^−1^, respectively, showed the trend: **Rh-RGD** > cilengitide > c(RGDyK). More importantly, we found that the calculated TB-DFT dissociation energy yields a reliable quantification of the strength of target–ligand interactions, where the overall target–ligand binding energy also included other energy and entropy contributions, which added to the stability of the αvβ3–peptide adducts. This fact may also partially compensate for the interaction strength. Moreover, we envisioned that the differences in the target–ligand dissociation energies would be comparatively small, and assumed that cilengitide, c(RGDyK), and **Rh-RGD** were characterized by essentially similar affinities for the integrin receptor.

A different aspect emerged for the Au(i) conjugates. For the monomeric gold complex **Au-RGD**, in which one coordination site of Au(i) was occupied by the peptide, and the second by a halide ligand, further conformational analysis identified 16 low-energy structures, of which only five were found to be sterically compatible with the αvβ3 model. The calculated interaction energies of these **Au-RGD** conformers are significantly lower than those obtained for cilengitide, c(RGDyK), or **Rh-RGD**, ranging from 20 to 63 kcal mol^−1^. In the optimized geometries, while the RGDyK moiety of **Au-RGD** remained located in the RGD recognition region, the Au(i)-substituted pendant extended towards the solvent-exposed region, and did not reproduce key hydrogen-bonding interactions observed for c(RGDyK), particularly those associated with the d-tyrosine and arginine residues of the peptide and Asp126/Asp218. The weaker target–peptide interaction strength calculated in the **Au-RGD** adduct suggested that the binding to the integrin receptor of this metal complex may not be relevant to affect its biological profile.

Furthermore, the antiproliferative activity of the new conjugates was evaluated in a panel of cell lines with different integrin expression profiles: **MDA-MB435S** (human melanoma): αvβ3(++), αvβ5(++), and α5β1(−);^41^**MDA-MB-231** (human breast adenocarcinoma): αvβ3(+), αvβ5(++), and α5β1(+);^42^**A375** (human melanoma): αvβ3(++), αvβ5(+), and α5β1(+).^43^ Thus, our results demonstrated that, regardless of the integrin expression profile, peptide conjugation exerts no observable effect on the acute toxicity of the metal-containing moieties (EC_50_ > 50 μM, Table 1). This showed that **Rh-RGD** was non-toxic, as the Cp*Rh(iii) tris aqua complex, and previously reported SSTR2 Cp*Rh conjugates.^12^ Moreover, the Au(i) NHC derivatives investigated here further demonstrated no detectable cytotoxic effects, under the tested conditions. It should be noted that the respective precursor (1) was not sufficiently stable in aqueous media to allow a direct comparison.

**Table 1:** Antiproliferative activity of the OM-RGD compounds, expressed as EC_50_ (μM), against human cancer cell lines with varying integrin expression levels. Compounds were incubated with cells for 72 h. Cisplatin was also tested as a benchmark metallodrug

	Compound					
Cell line	c(RGDyK)	[Cp*Rh(H_2_O)_3_](OTf)_2_	**Rh-RGD**	**Au-RGD**	**Au-RGD**_**2**_	Cisplatin
MDA-MB435S melanoma: αvβ3(++), αvβ5(++), and α5β1(−)	>100	>100	>100	>50	>50	20.56 ± 1.80
MDA-MB-231 breast adenocarcinoma: αvβ3(+), αvβ5(++), and α5β1(+)	>100	>100	>100	∼50	>50	31.1 ± 4.0^a^
A375 melanoma: αvβ3(++), αvβ5(+), and α5β1(+)	>100	>100	>100	>50	>50	30.51 ± 2.60

a Ref. 44.

**Fig. 3:**
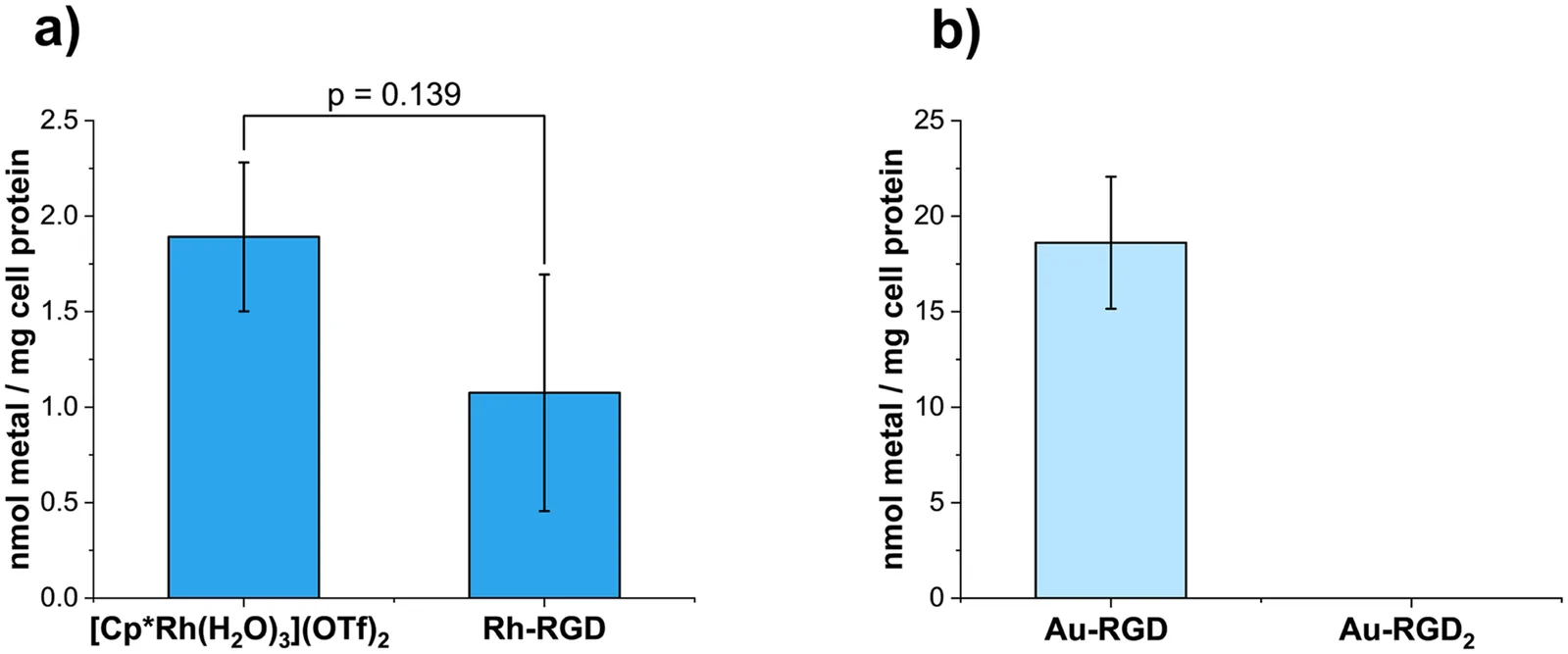
a) Rh cellular uptake after treatment with a [10 μM] Cp*Rh(iii) precursor or **Rh-RGD** conjugate. b) Au cellular uptake upon treatment [50 μM] with the Au(i)-cRGD conjugates. The metal content was measured in MDA-MB-231 cells after 24 hours of incubation. Statistical analysis was performed using an unpaired two-tailed Welch's *t*-test (unequal variances assumed; *n* = 3 biological replicates). *p*-Values > 0.05 were considered not statistically significant.

In determining whether conjugation to the peptide favored internalization of the OM-RGD derivatives, cellular uptake was evaluated in MDA-MB-231 cells using ICP-MS. Given their lack of antiproliferative effects over 72 h of incubation, the Cp*Rh(iii) complexes were administered at high concentrations (50 μM), whereas the Au(i) NHC compounds were administered at non-toxic concentrations (10 μM). Thus, the uptake values should be interpreted within each metal series, rather than as a direct quantitative comparison between Cp*Rh(iii) – and Au(i)-NHC-containing conjugates. Within the Cp*Rh series, intracellular Rh levels measured after treatment with **Rh-RGD** were not significantly different from those observed for the tris aqua complex, [Cp*Rh(H_2_O)_3_](OTf)_2_ (Fig. 3a), indicating that peptide conjugation does not improve Rh accumulation, under these conditions. Although **Rh-RGD** was calculated to display the most favourable interaction with the αvβ3 model, the biological results indicated that the computed energetic preference does not directly translate into effective cellular uptake. Such discrepancies may also arise from factors not represented in the static binding model, including attenuation of electrostatic interactions in the aqueous medium, steric or conformational effects at the cell surface, restricted receptor accessibility, or differences between initial receptor recognition and subsequent internalization.

Within the Au series, important differences were observed among the two gold(i) conjugates; the bis-peptide derivative **Au-RGD**_**2**_ showed no detectable gold accumulation (Fig. 3b). In contrast, the mono NHC **Au-RGD** conjugate displayed clear intracellular accumulation at the tested concentration. This difference further suggests that gold uptake does not rely on integrin recognition. Instead, it may involve alternative pathways enabled by the labile anionic ligand at the Au(i) center, which can be exchanged in biological media, particularly by thiol-containing biomolecules. Moreover, the overall hydrophilic character of the peptide compounds may also reduce their cellular uptake, *via* passive transport through cell membranes.

## Conclusions

We have reported three novel organometallic bioconjugates, **Rh-RGD**, **Au-RGD**, and **Au-RGD**_**2**_, obtained by conjugating Cp*Rh(iii) and Au(i) NHC organometallic fragments to the integrin-binding peptide c(RGDyK). These bioconjugates expand the limited family of bioorganometallic RGD derivatives by exploiting two distinct metal–peptide connection strategies: chemoselective Cp*Rh(iii) coordination to tyrosine and Au(i) NHC's wingtip conjugation through the lysine side chain.

Tight-binding DFT studies were performed to investigate the binding of OM-RGD compounds at the cilengitide binding pocket of the αvβ3 receptor. While the Cp*Rh-conjugation did not appreciably alter the recognition of the peptides in comparison with the observed cilengitide pose, the **Au-RGD** complex showcased weaker non-covalent interactions with the target residues. This outcome was further corroborated by the similarly high values of target–ligand dissociation energies for cilengitide, RGDyK and **Rh-RGD** compared to the appreciably lower value calculated for the **Au-RGD** adduct.

The biological evaluation showed that peptide conjugation did not substantially enhance the acute cytotoxicity of the metal fragments across the cell lines investigated, indicating that integrin targeting alone was insufficient to confer pronounced antiproliferative activity in this series. Nevertheless, the cellular uptake studies revealed marked differences between the metal bioconjugates. The **Rh-RGD** displayed intracellular accumulation comparable to that of the Cp*Rh(iii) tris aqua complex. Surprisingly, the **Au-RGD**_**2**_ complex showed no detectable uptake, while the monomeric **Au-RGD** conjugate was efficiently internalized by cancer cells. These results indicated that the recognition at the αvβ3 binding pocket plays a secondary role in the biological activity, while the major role in the biological profile of **Au-RGD** is more likely played by the ligand exchange reactivity of the Au(i) metal center.

Together with computational analyses, these findings suggested that cellular behavior was governed by a complex interplay between receptor recognition, steric accessibility, solvation effects, and metal-centered reactivity. Overall, this study highlights both the opportunities and limitations of c(RGD)-guided organometallic conjugates, and underscores the need to consider coordination chemistry alongside peptide–receptor affinity when designing targeted metallodrugs. Therefore, future studies should focus on tuning linker architecture, metal-fragment stability, and ligand-exchange properties, while incorporating competitive binding assays, mechanistic uptake experiments, and broader biological models to distinguish integrin-mediated transport from alternative uptake processes. Such investigations may provide a more rational basis for developing next-generation metallopeptides with improved selectivity, controlled intracellular delivery, and therapeutically relevant activity.

## Experimental section

### General

All the used reagents and solvents in this study were purchased from commercial sources and used without prior treatment unless indicated differently. Resins for SPPS were purchased from Sigma-Aldrich®. Amino acid building blocks were obtained from BLD Pharmatech GmbH.

Moisture- or oxygen-sensitive reactions were carried out using standard Schlenk techniques in a dry nitrogen atmosphere. Anhydrous solvents were transferred using syringes, and cannulas were flushed with nitrogen prior to use. Reactions at elevated temperatures were carried out on electric heating plates equipped with aluminium heat-on blocks, while ice–water baths were employed to cool mixtures below room temperature. Continuous stirring of reaction mixtures is assumed throughout. Drying over Na_2_SO_4_ was performed by gently mixing the solution with the drying agent, followed by filtration and rinsing of the filter cake. [Au(tht)Cl] was prepared following the procedure described by Uson *et al.*^45^ while the [Cp*Rh(H_2_O)_3_](OTf)_2_ precursor was synthesized as reported by Fish *et al.*^35^

NMR spectra were recorded either on a Bruker AV400 Ultra Shield or a Bruker 500HD spectrometer. In all cases, ^1^H-NMR and ^13^C chemical shifts (*δ*) of the signals are given in parts per million and referenced to residual protons in the deuterated solvents. Coupling constants (*J*) are given in Hz. MNova software (Mestrelab Research) was used for data processing. HR-ESI-MS spectra were recorded using a HESI Thermo Scientific Exactive Plus Orbitrap mass spectrometer in positive ion mode. Solutions of the compound in LC–MS grade solvents at concentrations ranging from 1 to 10 nM were prepared and analyzed. The spectra were acquired in positive-ion mode. Preparative HPLC purification was performed using a Thermo Scientific Dionex UltiMate 3000 system, equipped with an HPG-3200BX binary gradient pump and a VWD-3400RS UV/Vis detector. Spectra were recorded at 220 nm and 254 nm. Water (+0.01% TFA; solvent A) and acetonitrile (+0.01% TFA; solvent B), both of chromatography grade, served as eluents. Uvasol TFA was used as the elution solvent for spectroscopy. Preparative runs were performed at a flow rate of 10 mL min^−1^. The system was equipped with a MultoKrom column (100-5 C-18, 250 × 20 mm). Purified peptides were freeze-dried with a Büchi Lyovapor L-200. The high vacuum pump reached an average pressure of 0.06 mbar, and the ice condenser temperature was −56.6 °C.

### Synthesis of the cyclic-RGDyK peptide

The cyclic-RGDyK peptide was synthesized according to a literature procedure by Han *et al.*^34^ The cyclized peptide was synthesized in a 600 μmol scale. The isolated crude product was purified *via* RP-HPLC (0–20% B in 20 min); after lyophilization, c(RGDyK) was obtained as a white solid (170 mg, 274 μmol, 45%).

^**1**^ **H NMR** (400 MHz, D_2_O) *δ* [ppm] = 7.09–6.98 (m, 2H), 6.76–6.68 (m, 2H), 4.48 (t, *J* = 6.5 Hz, 1H), 4.40 (dd, *J* = 9.2, 7.0 Hz, 1H), 4.15 (t, *J* = 7.4 Hz, 1H), 3.96–3.81 (m, 3H), 3.73 (d, *J* = 15.1 Hz, 1H), 3.10 (t, *J* = 6.8 Hz, 2H), 2.91 (dd, *J* = 13.8, 6.9 Hz, 2H), 2.82–2.71 (m, 4H), 2.52–2.36 (m, 3H), 1.70 (q, *J* = 7.7 Hz, 2H), 1.51 (dtd, *J* = 43.3, 14.5, 7.2 Hz, 8H), 1.05 (s, 1H), 0.89 (s, 1H).

**HR-MS (ESI):** *m*/*z* = calculated for [C_27_H_42_N_9_O_8_]^+^: 620.3151, [M + H]^+^; [C_27_H_43_N_9_O_8_]^2+^: 310.6612, [M + 2H]^2+^; found: 620.3149, 310.6610.

### Synthesis of the Au(i)-bisNHC precursor

#### Perfluorophenyl 4-(bromomethyl)benzoate (a)

A solution of 4-(bromomethyl)benzoic acid (2.15 g, 10.0 mmol, 1.00 equiv.) and pentafluorophenol (1.84 g, 10.0 mmol, 1.00 equiv.) in ethyl acetate (30 mL) containing DMF (1 mL) was cooled to 0 °C. *N*,*N*′-Dicyclohexylcarbodiimide (DCC, 2.06 g, 10.0 mmol, 1.00 equiv.) was added, and the mixture was stirred at 0 °C for 1 hour before being allowed to warm to room temperature. After 2 hours, the precipitated dicyclohexylurea was removed *via* filtration. The filtrate was concentrated under reduced pressure and purified by column chromatography (*n*-hexane/ethyl acetate, 80/20, v/v) to afford compound (a) as a white solid (2.51 g, 6.59 mmol, 66%).

^**1**^ **H NMR** (400 MHz, CDCl_3_): *δ* [ppm] = 8.18 (d, 2H, ^3^*J*_HH_ = 8.0 Hz), 7.57 (d, 2H, ^3^*J*_HH_ = 8.0 Hz), 4.54 (s, 2H); ^**13**^**C NMR** (101 MHz, CDCl_3_): *δ* [ppm] = 162.2, 144.9, 142.7, 140.2, 139.4, 136.9, 131.4, 129.7, 126.9, 31.8; ^**19**^**F NMR** (376 MHz, CDCl_3_): *δ* [ppm] = −(151.67–153.36) (m, 2F), −157.73 (t, *J* = 21.7 Hz, 1F), −(161.36–162.92) (m, 2F).

#### 3-(4-((Perfluorophenoxy)carbonyl)benzyl)-1-phenyl-1*H*-imidazol-3-ium bromide (b)

A solution of compound (a) (1.0 g, 2.62 mmol, 1.00 equiv.) in anhydrous THF (10 mL) was added dropwise to a solution of 1-phenylimidazole (332 μL, 2.62 mmol, 1.00 equiv.) in anhydrous THF (10 mL). The reaction mixture was refluxed for 24 hours, resulting in the formation of a white precipitate. The solvent was decanted, and the precipitate was washed with THF (3 × 10 mL) and dried under vacuum to afford the product (b) as a white solid (0.97 g, 1.85 mmol, 70%).

^**1**^ **H NMR** (400 MHz, CDCl_3_) *δ* [ppm] = 11.06 (s, 1H), 8.15 (d, 2H, *J* = 7.8 Hz), 7.99–7.84 (m, 3H), 7.78–7.64 (m, 3H), 7.57–7.41 (m, 3H), 6.08 (s, 2H); ^**13**^**C NMR** (101 MHz, CDCl_3_) *δ* [ppm] = 162.0, 140.1, 136.0, 134.4, 131.7, 130.8, 130.6, 130.1, 127.9, 123.5, 121.9, 120.9, 52.8; ^**19**^**F NMR** (376 MHz, DMSO) *δ* [ppm] = −(153.43–153.70) (m, 2F), −157.32 (t, *J* = 23.2 Hz, 1F), −(162.02–162.46) (m, 2F).

#### Bis(1-(4-((perfluorophenoxy)carbonyl)benzyl)-3-phenyl-2,3-dihydro-1*H*-imidazol-2-yl)gold (1)

Compound (b) (200 mg, 0.38 mmol, 1.00 equiv.) and Cs_2_CO_3_ (147 mg, 0.76 mmol, 2.00 equiv.) were suspended in anhydrous dichloromethane (16 mL). The resulting orange suspension was stirred at room temperature for 2 hours. Afterward, a solution of [AuCl(tht)] (122 mg, 0.38 mmol, 1.00 equiv.) in anhydrous dichloromethane (5 mL) was added dropwise, and the reaction mixture was stirred overnight at room temperature in the dark. The suspension was filtered through celite, and the filtrate was concentrated under reduced pressure. Diethyl ether (35 mL) was added, and the resulting off-white suspension was filtered. The solid residue was washed with diethyl ether (3 × 20 mL) and dried *in vacuo* to afford (1) as a beige solid (195 mg, 179 mmol, 47%).

^**1**^ **H NMR** (400 MHz, CDCl_3_) *δ* [ppm] = 8.25 (d, 4H, *J* = 8.2 Hz), 7.73–7.66 (m, 4H), 7.63–7.55 (m, 4H), 7.54–7.46 (m, 6H), 7.27 (d, 2H, *J* = 1.8 Hz) 7.10 (d, 2H, *J* = 1.8 Hz), 5.63 (s, 4H); ^**13**^**C NMR** (101 MHz, CDCl_3_) *δ* [ppm] = 174.9, 162.1, 141.7, 138.8, 131.7, 130.0, 129.6, 128.7, 127.6, 124.8, 122.7, 120.9, 55.1.

**HR-MS-ESI:** *m/z* calc. for [C_46_H_26_AuF_10_N_4_O_4_]^+^ 1085.1460 [M–Br]^+^, found 1085.1432.

### Synthesis of the Rh(iii) bioconjugate Rh-RGD

The Cp*Rh-conjugate was synthesized according to a literature procedure from Fish *et al.*^12^ Both c(RGDyK) and [Cp*Rh(H_2_O)_3_](OTf)_2_ were dissolved separately in 2.5 mL of water, and the pH was adjusted to 5.1 for both solutions. The Cp*Rh tris aqua solution was then added to the peptide solution, and the reaction mixture was stirred for 48 hours at room temperature. The crude reaction mixture was filtered through a syringe filter before being subjected to RP-HPLC (0–10% B in 20 min). After lyophilization, **Rh-RGD** was obtained as an orange solid (8.00 mg, 6.92 μmol, 42%).

**HR-MS (ESI):** *m*/*z* = calculated for [C_37_H_56_N_9_O_8_Rh]^2+^: 428.6648, [M + H − 2OTf]^2+^; [C_37_H_55_N_9_O_8_Rh]^+^: 856.3223, [M − H − 2OTf]^+^; found: 428.6620, 856.3179.

### Synthesis of the Au(i) bioconjugates Au-RGD and Au-RGD_2_

The peptide c(RGDyK) (10.0 mg, 16.1 μmol, 1.00 equiv.) was dissolved in anhydrous DMF (2.0 mL). DIPEA (14.0 μL, 80.7 μmol, 5.00 equiv.) was added, and the solution was stirred for 10 minutes at room temperature. A solution of (1) (9.0 mg, 8.0 μmol, 0.50 equiv.) in anhydrous DMF (0.5 mL) was then added dropwise. The reaction mixture was stirred at room temperature for 24 hours. The mixture was passed through a syringe filter. Diethyl ether (35 mL) was added, and the resulting white suspension was centrifuged. The solid residue was washed with anhydrous methanol (5 × 20 mL) and dried *in vacuo* before being subjected to RP-HPLC (10–45% B in 20 min). After lyophilization, **Au-RGD** and **Au-RGD**_**2**_ were obtained as white solids (**Au-RGD**: 1.51 mg, 1.36 μmol, 8%/**Au-RGD**_**2**_: 1.00 mg, 0.50 μmol, 3%).

**HR-MS (ESI): Au-RGD:** *m*/*z* calc. for [C_44_H_54_AuN_11_O_9_]^2+^: 538.6680 [M + H–Cl]^2+^; [C_44_H_54_AuN_11_O_9_Cl]^+^: 1112.3455 [M + H]^+^; found: 538.6676, 1112.3475.

**Au-RGD**_**2**_ **:** *m*/*z* calc. for [C_88_H_108_AuN_22_O_18_]^3+^ 652.5954 [M + 2H]^3+^; found: 652.5923.

### Computational methods

The αvβ3 model was extrapolated from the deposited structure of the αvβ3-cilengitide adduct (PDB: 1L5G).^36^ The target model was obtained in the Maestro workspace^46^ by selecting the protein segments within 12.0 Å from the ligand, *i.e.* three αv segments 146–155, 172–182, and 209–222, and five β3 segments 118–128, 154–161, 174–189, 210–222, and 248–255. All segments were capped by either the *N*-acetyl group (N-termini) or methyl-amide group (C-termini). The Protein Preparation tool in Maestro was used to add hydrogen atoms by assuming the physiological pH = 7.4. The co-crystallized structure of cilengitide was also prepared in the Maestro workspace and used as the reference ligand binding pose. The 3D models of c(RGDyK), **Rh-RGD** and **Au-RGD** were built in the Maestro workspace. To enhance the optimization of their poses within the αvβ3 model, the structures of c(RGDyK), **Rh-RGD** and **Au-RGD** were rototranslated in the Maestro workspace to superimpose their cyclic peptide moieties on the cilengitide structure. The obtained geometries of the cilengitide-, c(RGDyK)-, and **Rh-RGD**-αvβ3 adducts were then optimized at the tight-binding DFT level of theory implemented using xTB software (version 6.7.1).^32^ All models were described using the GFN2-xTB scheme,^33,34^ and by employing the *alpb* algorithm to model the effect of the aqueous bulk.^35^ To control the geometrical artifacts possibly caused by truncation, the alpha carbon atoms of all protein segments were constrained by the application of a harmonic potential (xTB setting: *force constant* = 1.0000). Therefore, the *Fast Inertial Relaxation Engine* algorithm^47^ was used to drive the geometry optimization by assuming 0.5000000 × 10^−5^ h and 0.1000000 × 10^−2^ h bohr^−1^ as energy and gradient convergence thresholds, respectively. An alternative approach was adopted to calculate the binding pose in the **Au-RGD**-αvβ3 adduct because of the high flexibility of this ligand. Thus, the conformational space of the **Au-RGD** ligand was preliminarily explored using metadynamics^48^ calculations (GFN2, alpb = water).^37–39^ The ligand was simulated using a Berendsen thermostat at 298.15 K for 50 ps (a time step of 4 fs) and adopting the SHAKE constraining scheme. The metadyamics parameters controlling the collective variable potential were set *kpush* = 0.5, *alpha* = 1.0, and *update* = 10 steps. The close-to-minimum structures dumped from the metadynamics trajectories were optimized (GFN2, alpb = water)^37–39^ and energetically re-ranked. The 50 lowest energy conformers were then docked in the αvβ3 model using the docking submodule of xTB, based on the automated computational interaction site screening (aISS) workflow,^49^ to rapidly assess their steric prerequisites in the fitting of the cilengitide binding pocket. Eventually, only 16 conformations were found to sterically access the αvβ3 binding site, and their corresponding target–ligand structures were optimized with the same approach adopted for cilengitide-, c(RGDyK)-, and **Rh-RGD**- αvβ3 adducts. The target–ligand dissociation energies were calculated by the xTB optimization of the target and ligand structures for each of the cilengitide-, c(RGDyK)-, **Rh-RGD**- and **Au-RGD**-αvβ3 adducts, through the simple formula:*E*_diss_ = *E*_target–ligand_ − (*E*_target_ − *E*_ligand_)

### Cell culture maintenance

Human A375 melanoma cells were obtained from Cell Line Service (CLS, Eppelheim, Germany), while human MDA MB-231 breast cancer cells and human MDA-MB-435S melanoma cells were obtained from the Helmholtz Institute for Infection Research (HZI, Braunschweig, Germany). Cells were cultured in Dulbecco's Modified Eagle's Medium (DMEM; high glucose, pyruvate, no glutamine), supplemented with 10% (v/v) heat-inactivated fetal bovine serum (qualified, South American origin), 1% (v/v) gentamicin sulphate solution (50 mg mL^−1^; final concentration 50 mg L^−1^), and 1% (v/v) l-glutamine solution (200 mM; final concentration 2 mM). Cells were passaged twice weekly. All reagents were purchased from Gibco™ (Thermo Scientific), unless otherwise stated, and solvents were obtained from Sigma-Aldrich. Ultrapure water (18.2 MΩ cm at 25 °C) was produced using a BerryPure® mini system (Berrytec).

### Antiproliferative activity

A375 cells (7000 cells per well) or MDA-MB-231 cells (8000 cells per well) or MDA-MB-435S (5000 cells per well) were seeded into flat-bottomed 96-well microtiter plates and incubated at 37 °C in a humidified atmosphere containing 5% CO_2_ for 24 h. Stock solutions of the compounds were freshly prepared as suspensions in PBS (pH 7.4) and diluted in the respective culture medium to obtain graded concentrations. Final concentrations ranged from 0 to 100 μM for the free peptide and Rh precursor, and from 0 to 50 μM for the gold peptides. After 24 h, the cells were treated with 100 μL of each compound solution and incubated for 72 h. Subsequently, the culture medium was replaced with 100 μL of freshly prepared and sterile-filtered MTT solution (0.5 mg mL^−1^ in PBS), and the plates were incubated for 90 min. Absorbance was measured using an Infinite 200 PRO® plate reader (Tecan) at 570 nm (MTT signal) and 690 nm (background) and expressed as the difference between the two readings. EC_50_ values (half-maximal effective concentrations) were calculated as the concentration required to inhibit cell proliferation by 50% compared to untreated controls (PBS in DMEM). Data represent the mean of three independent experiments.

### Determination of cellular uptake

Cellular uptake was evaluated in the MDA-MB-231 breast cancer cell line. Cells were grown to approximately 80% confluence and then treated for 24 h with the compounds at the following concentrations: 50 μM for the Rh(iii) precursor and **Rh-RGD**, and 10 μM for the gold bioconjugates. After treatment, cell monolayers were washed with cold PBS, detached, lysed, and subsequently mineralized in Teflon microwave vessels using high-purity nitric acid (TraceSELECT® Ultra, Au ≤ 0.01 μg kg^−1^, Rh ≤ 0.01 μg kg^−1^; Sigma-Aldrich) using a CEM Discover SP-D Plus Microwave Digestion System. Following digestion and cooling, the metal content was quantified by inductively coupled plasma mass spectrometry (ICP-MS) using an Agilent 7900 ICP-MS instrument.

## Conflicts of interest

There is no conflict of interest to declare.

## Supplementary Material

MD-OLF-D6MD00713A-s001

## Data Availability

The data supporting this article, including additional experimental procedures, characterisation data, and additional figures and tables have been included as part of the supplementary information (SI). Supplementary information is available. See DOI: https://doi.org/10.1039/d6md00713a.
