# Supplementary material for: Bioactivity studies of organometallic Cp*Rh(iii) and Au(i) NHC complexes bioconjugated to integrin binding peptides
Source: RSC Med Chem. 2026 Aug 31;17(10):4957–66. doi: 10.1039/d6md00713a (PMC13598753; doi:10.1039/d6md00713a)
Supplement: Supplementary file 1 [file MD-017-D6MD00713A-s001.pdf]

Supplementary Information (SI) for RSC Medicinal Chemistry.
This journal is © The Royal Society of Chemistry 2026

Supporting Information

Bioactivity Studies of Organometallic Cp*Rh(III) and Au(I) NHC Complexes Bioconjugated to Integrin Binding Peptides

Felix A. Böhm, [a,b] Miriam Caviglia, [a,c] Bernd Schulz, [a] Claudia Schmidt, [a] Roland A. Fischer,[b] Richard H. Fish,[d] Iogann Tolbatov, [e] Alessandro Marrone, [f,*] Guillermo Moreno-Alcántar, [a,g,*] and Angela Casini [a,*]

[a] *Chair of Medicinal and Bioinorganic Chemistry, Department of Chemistry, School of Natural Sciences, Technical University of Munich, Garching, Germany*

[b] *Chair of Inorganic and Metal-Organic Chemistry, Department of Chemistry, School of Natural Sciences, Technical University of Munich, Garching, Germany*

[c] *School of Science and Technology, Chemistry Division (ChIP), University of Camerino, Camerino, Italy*

[d] *Lawrence Berkeley National Laboratory, University of California, Berkeley, California, United States*

[e] *Department of Chemical, Physical, Mathematical and Natural Sciences, University of Sassari, Via Vienna 2, 07100 Sassari, Italy*

[f] *Department of Pharmacy, University "G. D'Annunzio" of Chieti-Pescara, Via dei Vestini 31, 66100 Chieti, Italy*

[g] *Center for Cooperative Research in Biomaterials (CIC biomaGUNE), Basque Research and Technology Alliance (BRTA), Paseo de Miramon 194, 20014, Donostia-San Sebastián, Spain*

*Corresponding authors: amarrone@unich.it; gmorenoalcantar@cicbiomagune.es; angela.casini@tum.de

Table of Contents:

1. Experimental 3
  1.1 Materials and Methods 3
    1.1.1 Solvents and Reagents 3
    1.1.2 Instrumentation and Standard Procedures 3
    1.1.3 Synthesis of the cyclic-RGDyK peptide: 4
  1.2 Synthetic Protocol 6
2. NMR Spectra 12
3. HR-MS Spectra 17
4. Tables 20
5. References 20

1. Experimental

1.1 Materials and Methods

1.1.1 Solvents and Reagents

All the used reagents and solvents in this study were purchased from commercial sources and used without prior treatment unless indicated differently. Resins for SPPS were purchased from Sigma-Aldrich®. Amino acid building blocks were obtained from BLD Pharmatech GmbH.

Moisture- or oxygen-sensitive reactions were carried out using standard SCHLENK techniques in a dry nitrogen atmosphere. Anhydrous solvents were transferred using syringes, and cannulas were flushed with nitrogen prior to use. Reactions at elevated temperatures were carried out on electric heating plates equipped with aluminium heat-on blocks, while ice-water baths were employed to cool mixtures below room temperature. Continuous stirring of reaction mixtures is assumed throughout. Drying over Na_2SO_4 was performed by gently mixing the solution with the drying agent, followed by filtration and rinsing of the filter cake.

[Au(tht)Cl] was prepared following the procedure described by Uson *et al.*[1] Natriumtetrachloroaurat (III) dihydrate (1.1 g, 2.78 mmol, 1.00 equiv.) was dissolved in a water/ethanol mixture (18 mL, 1:5 v/v). Tetrahydrothiophene (tht) (514 µL, 5.81 mmol, 2.10 equiv.) was added dropwise, and the resulting mixture was stirred at room temperature for 2 hours. The formed precipitate was collected by filtration and washed with cold ethanol (1 × 10 mL) and hexane (6 × 40 mL). The product was dried under vacuum to afford a white solid (749 mg, 2.34 mmol, 84%).

The **[Cp*Rh(H_2O)$_3$](OTf)$_2$** precursor was synthesized as reported by Fish *et al.*[2], [Cp*RhCl_2]$_2$ (200 mg, 0.32 mmol, 1.00 equiv.) and silver trifluoromethanesulfonate (334 mg, 1.30 mmol, 4.00 equiv.) were dissolved in anhydrous dichloromethane (40 mL) and stirred at room temperature for 6 hours. The resulting suspension was filtered to remove precipitated AgCl. The filtrate was layered with water (40 µL, 2.22 mmol, 7.00 equiv.) and left undisturbed at room temperature for 12 hours, affording orange crystals after filtration (87 mg, 0.15 mmol, 46%).

1.1.2 Instrumentation and Standard Procedures

NMR:

NMR spectra were recorded either on a Bruker AV400 Ultra Shield or a Bruker 500HD spectrometer. In all cases, ^{1}H-NMR and ^{13}C chemical shifts (δ) of the signals are given in parts per million and referenced to residual protons in the deuterated solvents. Coupling constants (*J*) are given in Hz. MNova software (Mestrelab Research) was used for data processing.

HR-ESI-MS:

HR-ESI-MS spectra were recorded on a HESI Thermo Scientific Exactive Plus Orbitrap Mass Spectrometer in positive ion mode. Solutions of the compound in LC-MS grade solvents at concentrations ranging from 1 to 10 nM were prepared and analyzed. The spectra were acquired in positive mode.

Reversed-Phase High-Performance Liquid Chromatography (RP-HPLC):

Preparative HPLC purification was performed on a Thermo Scientific Dionex UltiMate 3000 system, equipped with an HPG-3200BX binary gradient pump and a VWD-3400RS UV/Vis detector. Spectra were recorded at 220 nm and 254 nm. Water (+0.01% TFA; solvent A) and acetonitrile (+0.01% TFA; solvent B), both of chromatography grade, served as eluents. Uvasol TFA was used as the elution solvent for spectroscopy. Preparative runs were performed at a flow rate of 10 mL/min. The system was equipped with a column from MultoKrom (100-5 C-18 of size 250 × 20 mm).

Lyophilization:

Purified peptides were freeze-dried with a Büchi Lyovapor L-200. The high vacuum pump reached an average pressure of 0.06 mbar, and the ice condenser temperature was -56.6 °C.

1.1.3 Synthesis of the cyclic-RGDyK peptide:

The cyclic-RGDyK peptide was synthesized according to a modified literature procedure by Han *et al*.[3]

General Procedure 1: Loading of 2-CTC-resin.

Peptide synthesis was carried out in PP-reactor syringes with PE frits (MultiSynTech GmbH) in 20 mL syringes, each locked with PP plugs, using 2-chlorotrityl chloride (2-CTC) resin (1.6 mmol/g). The reaction was performed on a 600 µmol scale for research purposes, following the standard Fmoc strategy. Before loading, the resin was swelled with anhydrous DCM (5 mL) for 10 minutes. All reagents were prepared freshly before each synthesis. Fmoc-Gly-OH (1.20 equiv.) was attached to the resin with DIPEA (2.50 equiv.) in anhydrous DCM (5 mL) for 2 hours at room temperature. The remaining 2-chlorotrityl chloride groups were capped by adding a mixture of MeOH/DIPEA (5:1; v/v; 5 mL) for 15 minutes. The resin was washed with DCM (3×) and DMF (3×). The loading capacity was estimated to be 1.6 mmol/g (100%).

General Procedure 2: On-Resin Fmoc Deprotection.

The resin-bound Fmoc peptide was treated with 20% (v/v) piperidine (3 mL) in DMF once (1 × 15min) and subsequently washed with DMF (3×), DCM (3×), and DMF (3×).

General Procedure 3: Standard Amino Acid Coupling.

A solution of Fmoc-AA-OH (2.00 equiv.), O-(7-azabenzotriazol-1-yl)-N,N,N',N'tetramethyluronium-hexafluorphosphate (HATU) (1.90 equiv.), 1-hydroxy-7-azabenzotriazole (HOAt; 2.00 equiv.), and DIPEA (5.00 equiv.) in DMF (5 mL) were added to the resin-bound free amine peptide and shaken for 60 minutes at room temperature. The resin was then washed with DMF (3×), DCM (3×), and DMF (3×).

General Procedure 4: Cleavage of Linear Peptides from the Resin.

For complete cleavage of the linear side chain protected peptide from the resin, the resin was treated with a solution of DCM and hexafluoroisopropanol (HFIP; 4:1; v/v) twice for 15 minutes at room temperature (2× 6 mL). The solvent was then evaporated under reduced pressure. The crude material was directly used for the next step without further purification.

General Procedure 5: Peptide Cyclization.

To a solution of linear peptide in DMF (2 mM peptide concentration, 300 mL) and $NaHCO_3$ (5.00 equiv.), diphenylphosphoryl azide (DPPA; 3.00 equiv.) was added at room temperature and stirred overnight. The solvent was concentrated, excess $NaHCO_3$ was filtered, and the remaining solvent was removed under reduced pressure (3× co-evaporated with toluene). The crude material was directly used for the next step without further purification.

General Procedures 6: Global Deprotection.

Cyclized peptides were stirred in a cleavage cocktail of TFA, water, and TIPS (95/2.5/2.5; v/v/v; 10 mL) at room temperature for 2 hours. The cleavage cocktail was concentrated to about 2 mL before the tenfold amount of 4 °C cooled diisopropyl ether was added for precipitation. The precipitate was then separated from the solution by centrifugation. The isolated crude product was purified via RP-HPLC (0-20% B in 20min) to obtain the cyclized peptide.

1.2 Synthetic Protocol

Perfluorophenyl 4-(bromomethyl)benzoate (a)

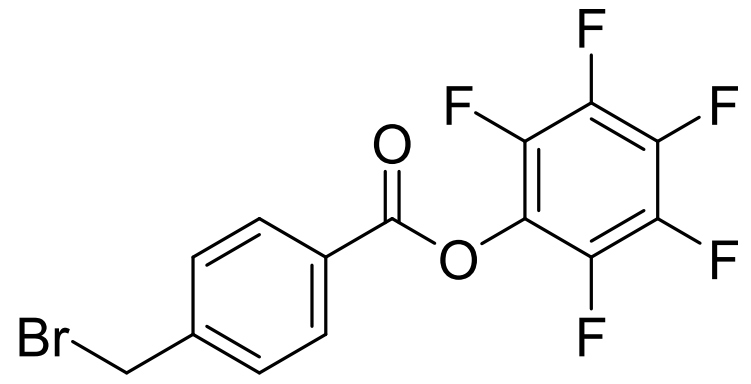

(a)

A solution of 4-(bromomethyl)benzoic acid (2.15 g, 10.0 mmol, 1.00 equiv.) and pentafluorophenol (1.84 g, 10.0 mmol, 1.00 equiv.) in ethyl acetate (30 mL) containing DMF (1 mL) was cooled to 0 °C. N,N′-Dicyclohexylcarbodiimide (DCC, 2.06 g, 10.0 mmol, 1.00 equiv.) was added, and the mixture was stirred at 0 °C for 1 hour before being allowed to warm to room temperature. After 2 hours, the precipitated dicyclohexylurea was removed via filtration. The filtrate was concentrated under reduced pressure and purified by column chromatography (n-hexane/ethyl acetate, 80/20, v/v) to afford compound **(a)** as a white solid. (2.51 g, 6.59 mmol, 66%).

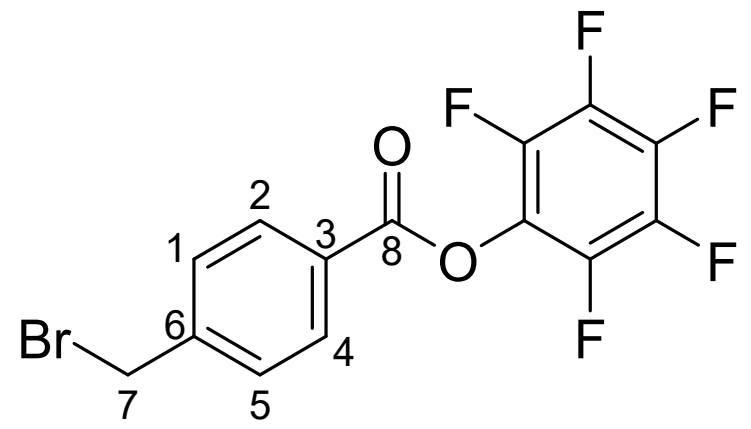

(a)

^{1}H NMR (400 MHz, $CDCl_3$): δ [ppm] = 8.18 (d, 2H, $^3J_{HH}$ = 8.0 Hz, H_2 & H_4), 7.57 (d, 2H, $^3J_{HH}$ = 8.0 Hz, H_1 & H_5), 4.54 (s, 2H, H_7); **^{13}C NMR** (101 MHz, $CDCl_3$): δ [ppm] = 162.2 (C_8), 144.9 (C_3), 142,7 (CF),140.2 (CF), 139.4 (CF), 136.9 (CF), 131.4 (C_2 & C_4), 129.7 (C_1 & C_5), 126.9 (C_6), 31.8 (C_7); **^{19}F NMR** (376 MHz, $CDCl_3$): δ [ppm] = −(151.67–153.36) (m, 2F), −157.73 (t, J = 21.7 Hz, 1F), −(161.36–162.92) (m, 2F).

3-(4-((perfluorophenoxy)carbonyl)benzyl)-1-phenyl-1H-imidazol-3-ium bromide (b)

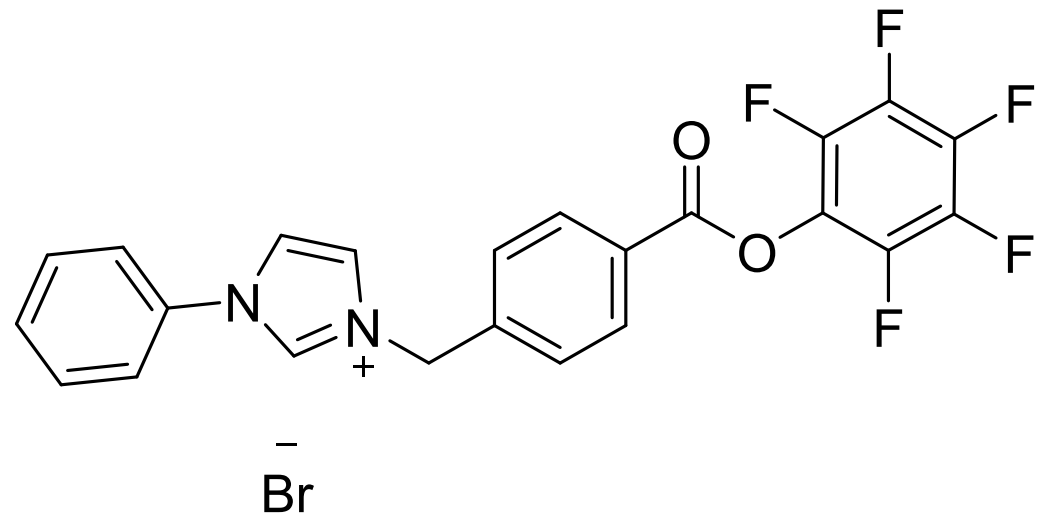

(b)

A solution of compound **(a)** (1.0 g, 2.62 mmol, 1.00 equiv.) in anh. THF (10 mL) was added dropwise to a solution of 1-phenylimidazole (332 µL, 2.62 mmol, 1.00 equiv.) in anh. THF (10 mL). The reaction mixture was refluxed for 24 hours, resulting in the formation of a white precipitate. The solvent was decanted, and the precipitate was washed with THF (3 × 10 mL) and dried under vacuum to afford the product **(b)** as a white solid (0.97 g, 1.85 mmol, 70%).

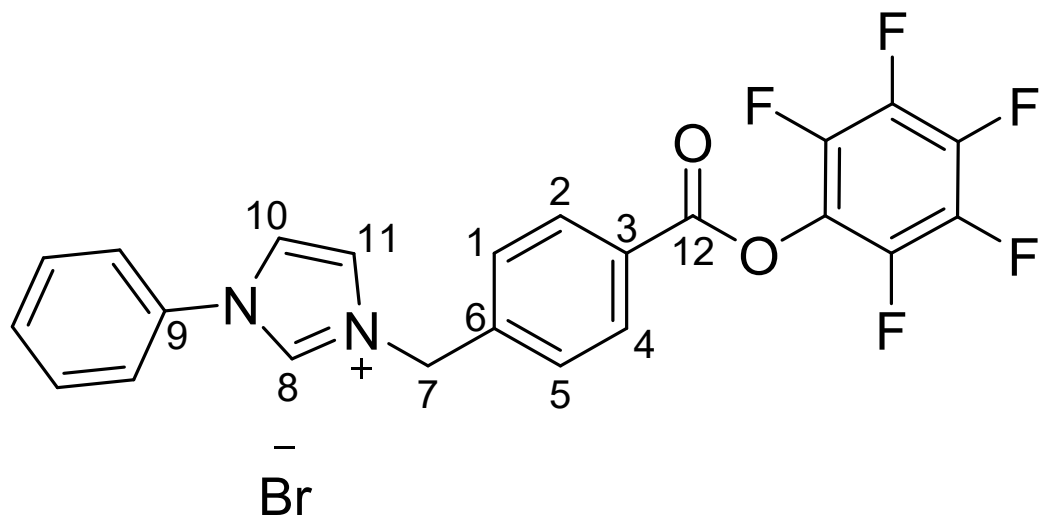

(b)

^{1}**H NMR** (400 MHz, $CDCl_3$) δ [ppm] = 11.06 (s, 1H, H_8), 8.15 (d, 2H, *J* = 7.8 Hz, H_2 &), 7.99 – 7.84 (m, 3H, $H_{arom.}$), 7.78 – 7.64 (m, 3H, H_1 &), 7.57 – 7.41 (m, 3H, $H_{arom.}$ & H_{10}), 6.08 (s, 2H, H_7); ^{13}**C NMR** (101 MHz, $CDCl_3$) δ [ppm] = 162.0 (C_8), 140.1 (C_3), 136.0 (C_9), 134.4 ($C_{arom.}$), 131.7 (C_2 & C_4), 130.8 (C_1 & C_5), 130.6 ($C_{arom.}$), 130.1 ($C_{arom.}$), 127.9 (C_6), 123.5 (C_{10}), 121.9 ($C_{arom.}$), 120.9 (C_{11}), 52.8 (C_7); ^{19}**F NMR** (376 MHz, DMSO) δ [ppm] = −(153.43–153.70) (m, 2F), −157.32 (t, *J* = 23.2 Hz, 1F), −(162.02–162.46) (m, 2F).

Bis(1-(4-((perfluorophenoxy)carbonyl)benzyl)-3-phenyl-2,3-dihydro-1H-imidazol-2-yl)gold (1)

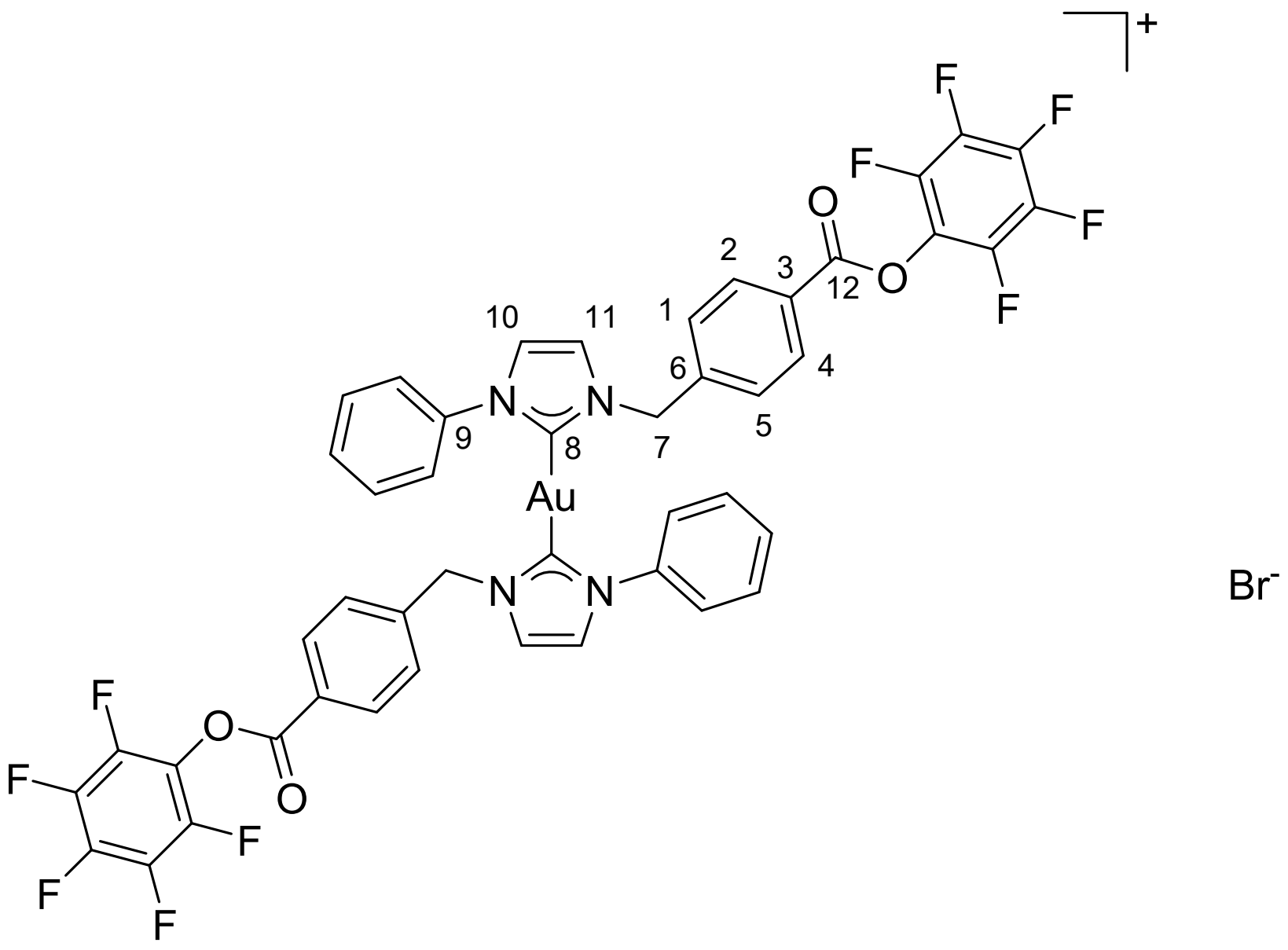

3

The compound **(b)** (200 mg, 0.38 mmol, 1.00 equiv.) and Cs_2CO_3 (147 mg, 0.76 mmol, 2.00 equiv.), were suspended in anh. dichloromethane (16 mL). The resulting orange suspension was stirred at room temperature for 2 hours. Afterward, a solution of [AuCl(tht)] (122 mg, 0.38 mmol, 1.00 equiv.) in anh. dichloromethane (5 mL) was added dropwise, and the reaction mixture was left to stir overnight at room temperature in the dark. The suspension was filtered through CELITE, and the filtrate was concentrated under reduced pressure. Diethyl ether (35 mL) was added, and the resulting grey suspension was filtered. The solid residue was washed with diethyl ether (3 × 20 mL) and dried *in vacuo* to afford **(1)** as a pale brown solid (195 mg, 179 mmol, 47%).

^{1}H NMR (400 MHz, $CDCl_3$) δ [ppm] = 8.25 (d, 4H, J = 8.2 Hz, H_2 &), 7.73 – 7.66 (m, 4H, $H_{arom.}$), 7.63 – 7.55 (m, 4H, $H_{arom.}$), 7.54 – 7.46 (m, 6H, H_1 & H_5 & $H_{arom.}$), 7.27 (d, 2H, J = 1.8 Hz, H_{10}) 7.10 (d, 2H, J = 1.8 Hz, H_{11}), 5.63 (s, 4H, H_7); **^{13}C NMR** (101 MHz, $CDCl_3$) δ [ppm] = 174.9, 162.1, 141.7, 138.8, 131.7, 130.0, 129.6, 128.7, 127.6, 124.8, 122.7, 120.9, 55.1.

HR-MS-ESI: *m/z* calc. for $[C_{46}H_{26}AuF_{10}N_4O_4]^+$ 1085.1460 $[M\text{-}Br]^+$, found 1085.1432.

Synthesis of c(RGDyK):

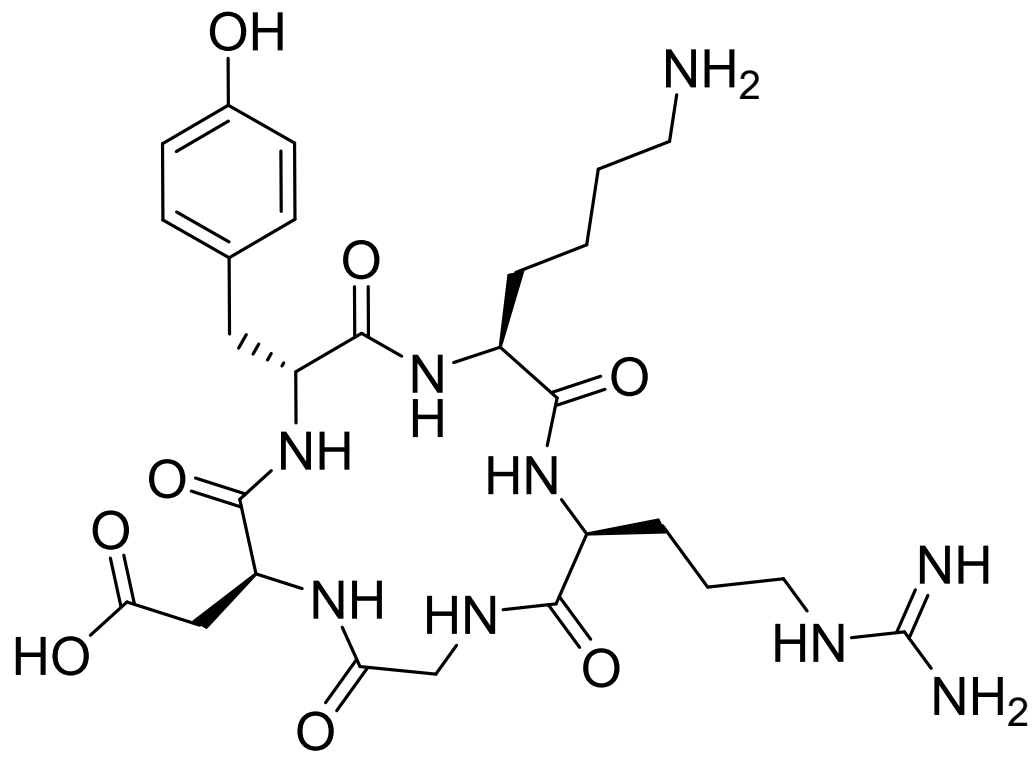

c(RGDyK)

The cyclized peptide was synthesized in a 600 µmol scale according to general procedures 1-6. The isolated crude product was purified via RP-HPLC (0-20% B in 20min), after lyophilization **c(RGDyK)** was obtained as a white solid (170 mg, 274 µmol, 45%).

^{1}H NMR (400 MHz, D_2O) δ [ppm] = 7.09 – 6.98 (m, 2H), 6.76 – 6.68 (m, 2H), 4.48 (t, *J* = 6.5 Hz, 1H), 4.40 (dd, *J* = 9.2, 7.0 Hz, 1H), 4.15 (t, *J* = 7.4 Hz, 1H), 3.96 – 3.81 (m, 3H), 3.73 (d, *J* = 15.1 Hz, 1H), 3.10 (t, *J* = 6.8 Hz, 2H), 2.91 (dd, *J* = 13.8, 6.9 Hz, 2H), 2.82 – 2.71 (m, 4H), 2.52 – 2.36 (m, 3H), 1.70 (q, *J* = 7.7 Hz, 2H), 1.51 (dtd, *J* = 43.3, 14.5, 7.2 Hz, 8H), 1.05 (s, 1H), 0.89 (s, 1H).

HR-MS (ESI): *m/z* = calculated for $[C_{27}H_{42}N_9O_8]^+$: 620.3151, $[M+H]^+$; $[C_{27}H_{43}N_9O_8]^{2+}$: 310.6612, $[M+2H]^{2+}$; Found: 620.3149, 310.6610.

Synthesis of Rh-RGD:

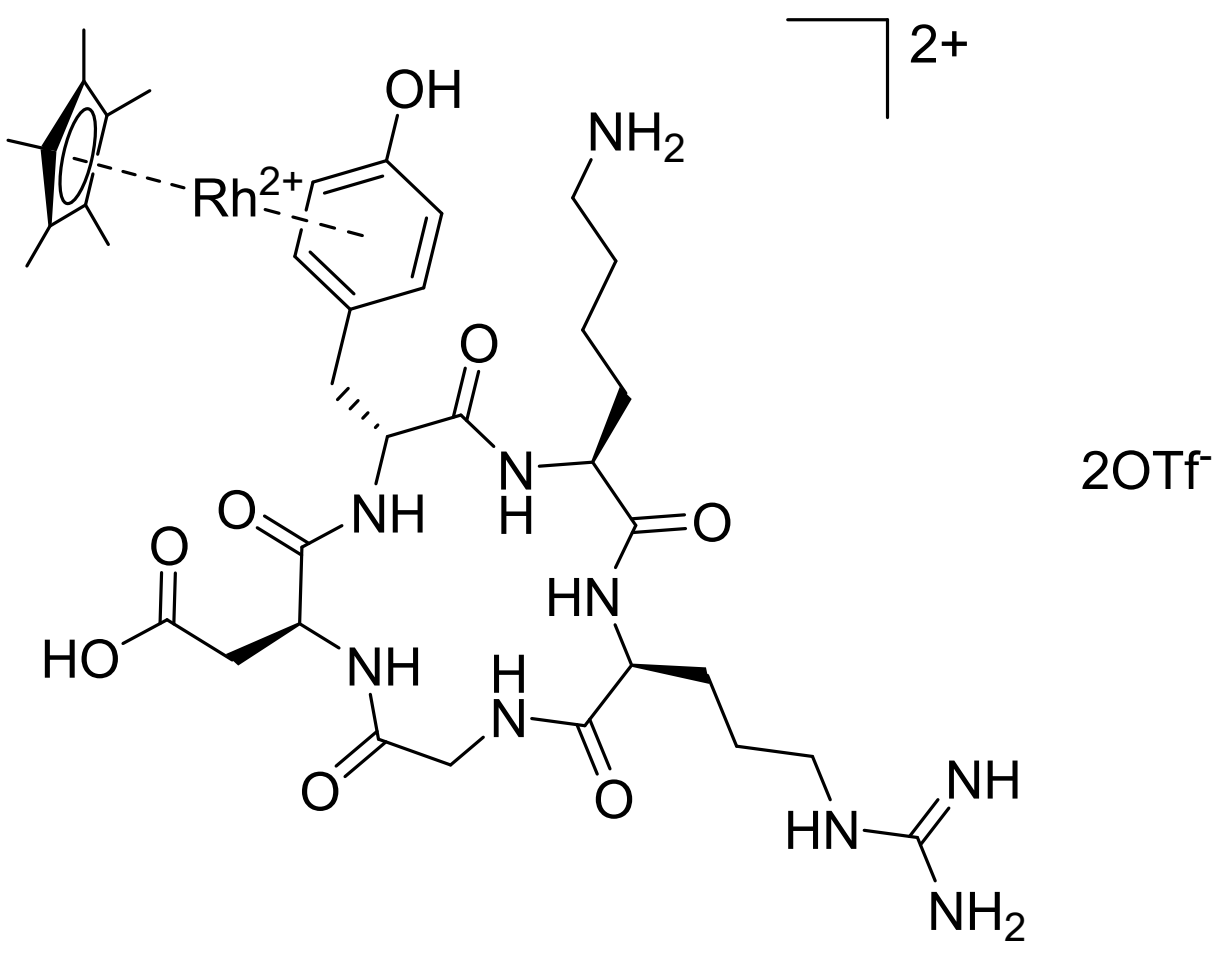

Rh-RGD

The Rh-conjugate was synthesized according to a modified literature procedure from Albada *et al*.[2] Both **c(RGDyK)** (10.0 mg; 16 mmol, 1.00equiv.) and **[Cp*Rh(H_2O)$_3$](OTf)$_2$** (14.3 mg; 24 mmol; 1.50 equiv) were dissolved separately in 2.5 mL water, and the pH was adjusted to 5.1 for both solutions. The rhodium solution was then added to the peptide solution, and the reaction mixture was stirred for 48 hours at room temperature. The crude reaction mix was filtered over a syringe filter before being subjected to RP-HPLC (0-10% B in 20min). After lyophilization, **Rh-RGD** was obtained as an orange solid (8.00 mg, 6.92 µmol, 42%).

HR-MS (ESI): *m/z* = calculated for $[C_{37}H_{56}N_9O_8Rh]^{2+}$: 428.6648, $[M+H-2OTf]^{2+}$; $[C_{37}H_{55}N_9O_8Rh]^{+}$: 856.3223, $[M-H-2OTf]^{+}$; Found: 428.6620, 856.3179.

Synthesis of the Au-conjugates: Au-RGD + Au-RGD$_2$:

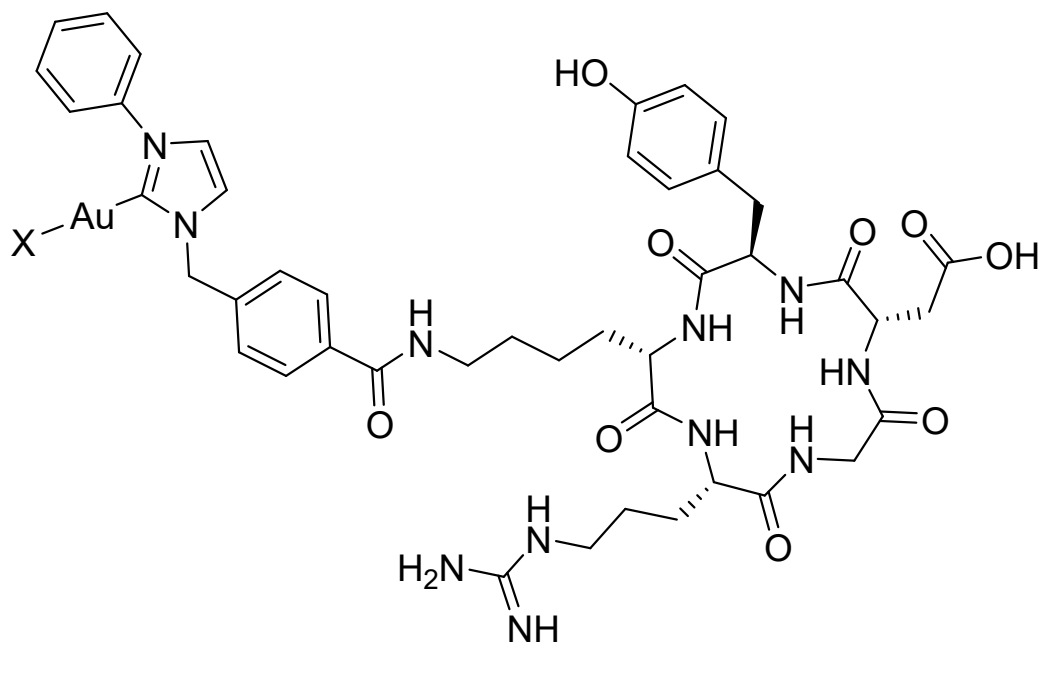

Au-RGD

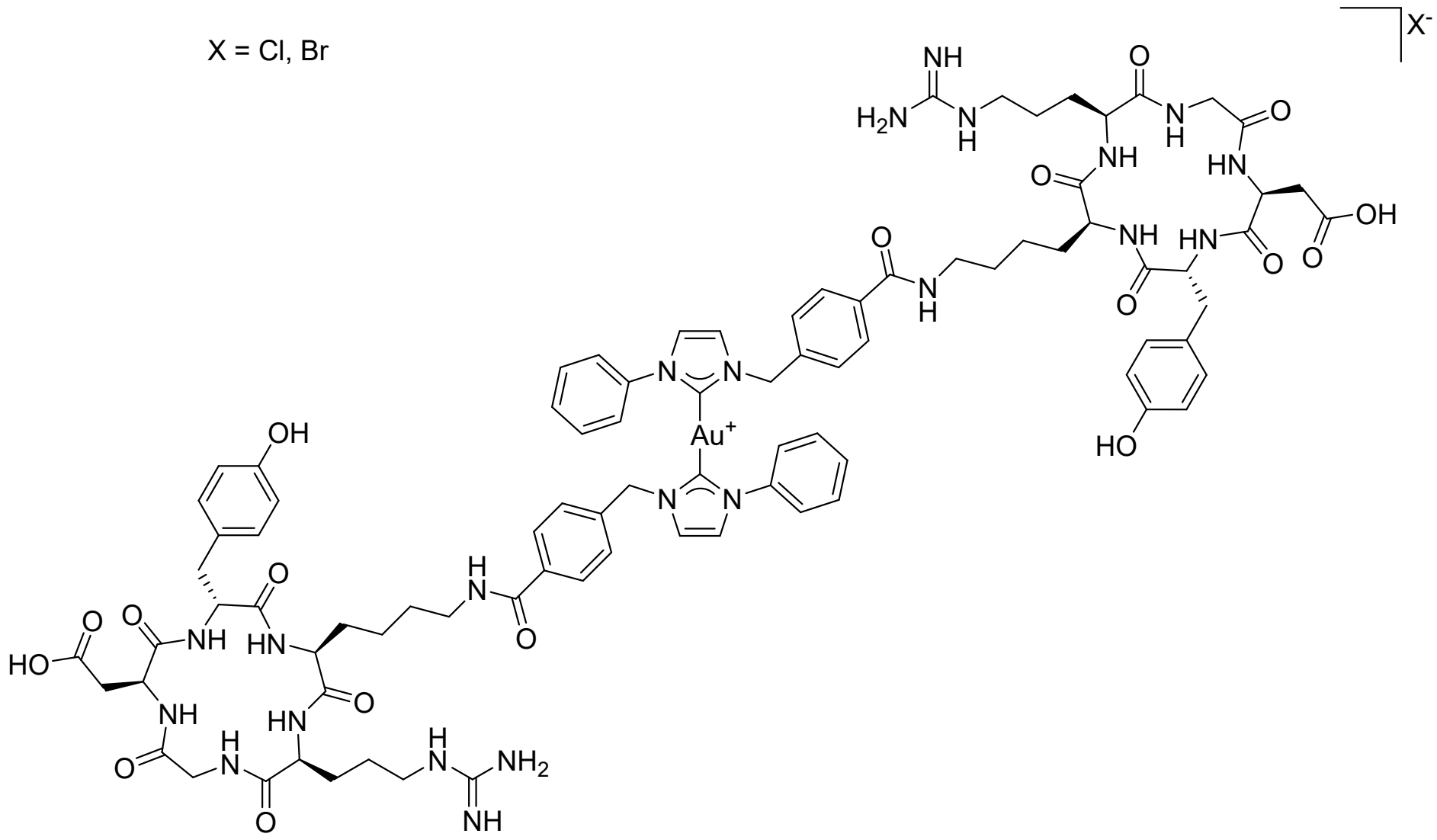

Au-RGD$_2$

The peptide **c(RGDyK)** (10.0 mg, 16.1 µmol, 1.00 equiv.) was dissolved in anh. DMF (2.0 mL). DIPEA (14.0 µL, 80.7 µmol, 5.00 equiv.) was added, and the solution was stirred for 10 minutes at room temperature. A solution of **(1)** (18.1 mg, 16.1 µmol, 1.00 equiv.) in anh. DMF (0.5 mL) was then added dropwise. The reaction mixture was stirred at room temperature for 24 hours. The mixture was passed through a syringe filter. Diethyl ether (35 mL) was added, and the resulting white suspension was centrifuged. The solid residue was washed with anh. methanol (5 × 20 mL) and dried in vacuo before being subjected to RP-HPLC (10-45% B in 20min). After lyophilization, **Au-RGD** and **Au-RGD$_2$** were obtained as white solids (**Au-RGD**: 1.51 mg, 1.36 µmol, 8% / **Au-RGD$_2$**: 1.00 mg, 0.50 µmol, 3%).

HR-MS (ESI):

6: m/z calc. for $[C_{44}H_{54}AuN_{11}O_9]^{2+}$: 538.6680 $[M+H-Cl]^{2+}$; $[C_{44}H_{54}AuN_{11}O_9Cl]^{+}$: 1112.3455 $[M+H]^{+}$; Found: 538.6676, 1112.3475.

7: m/z calc. for $[C_{88}H_{108}AuN_{22}O_{18}]^{3+}$ 652.5954 $[M+2H]^{3+}$; Found: 652.5923

2. NMR Spectra

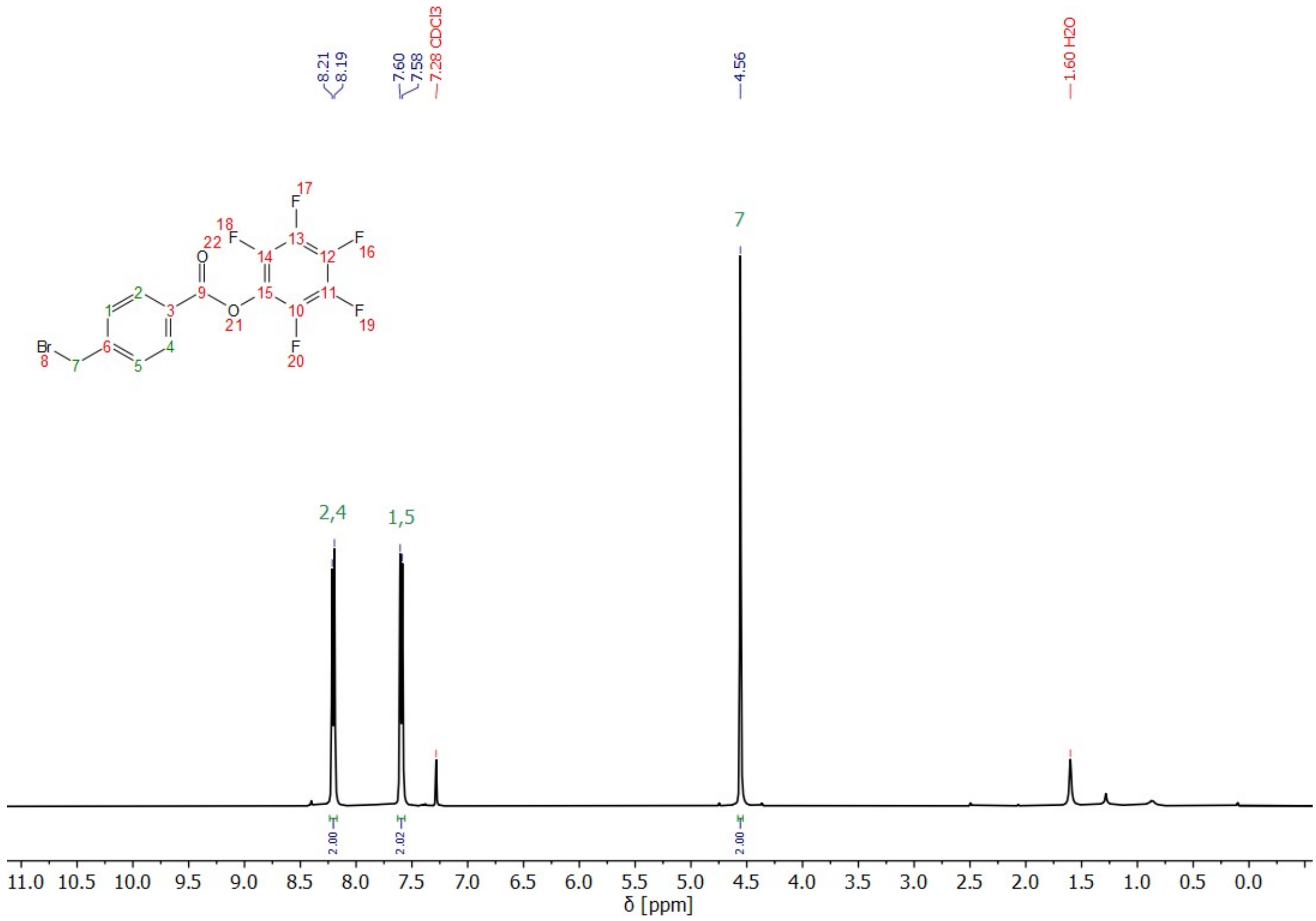

*Figure S1: ^{1}H-NMR spectra of compound **(a)** in $CDCl_3$ at 25°C, 400MHz.*

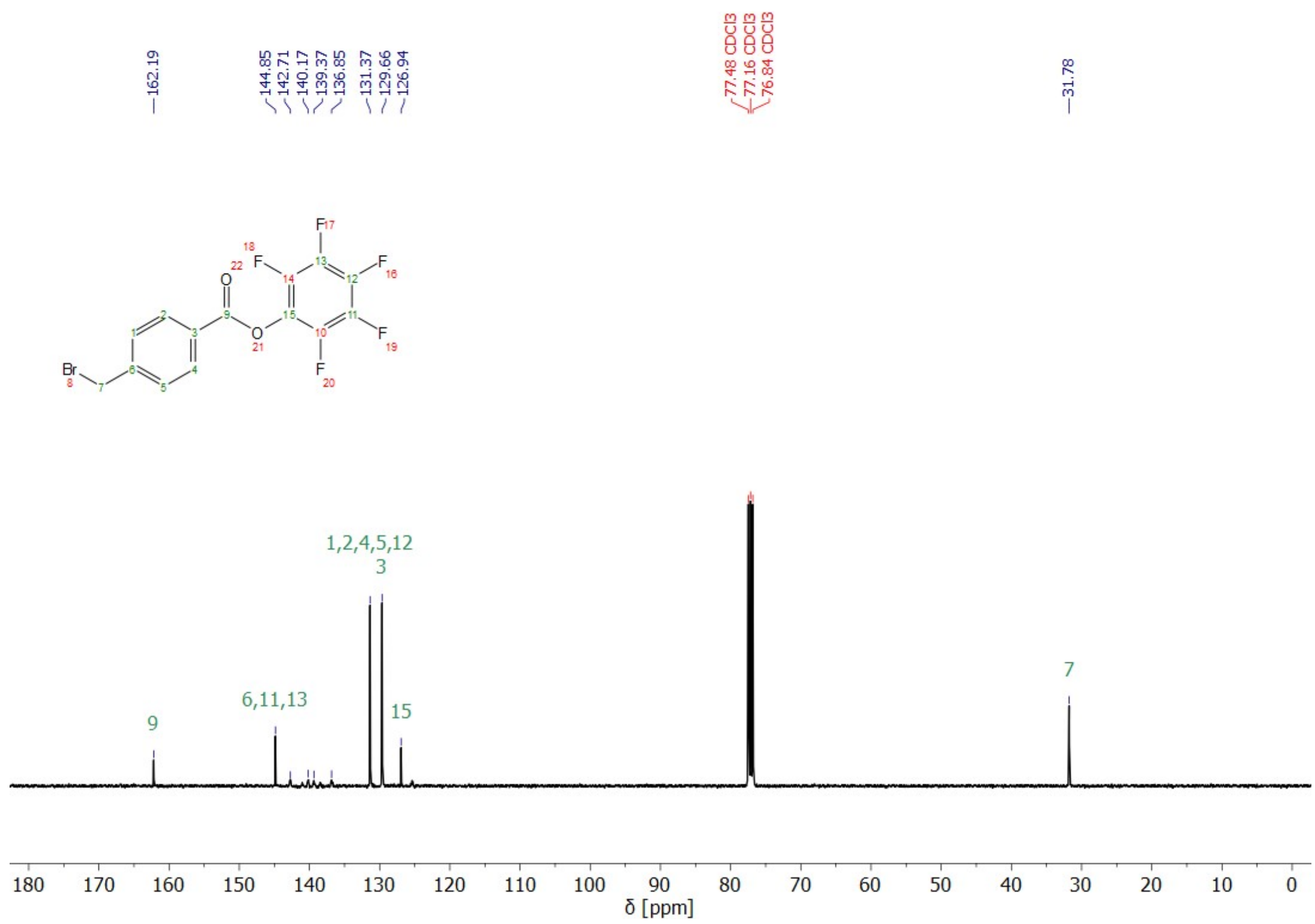

*Figure S2: ^{13}C-NMR spectra of compound **(a)** in $CDCl_3$ at 25°C, 400MHz.*

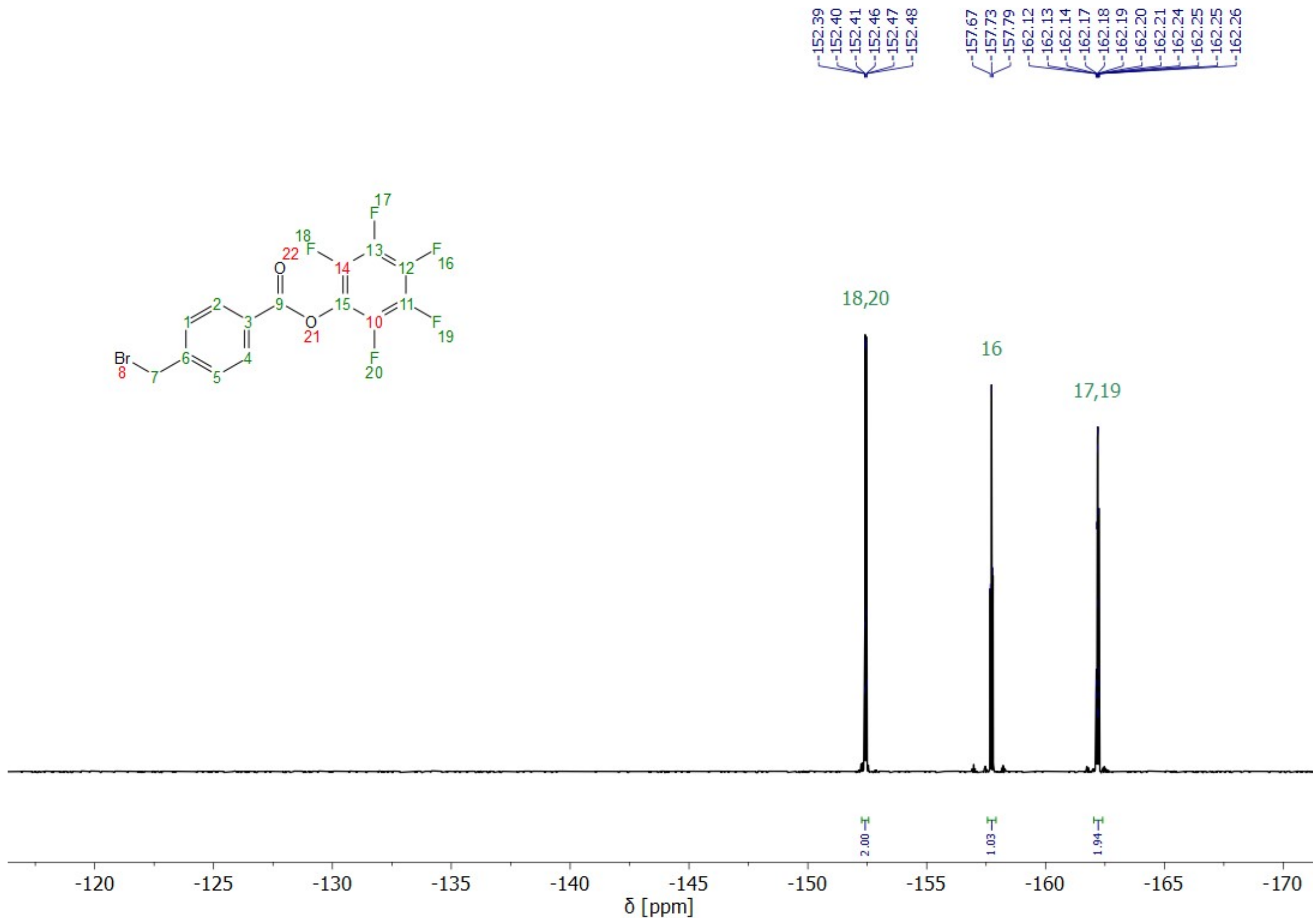

*Figure S3: ^{19}F-NMR spectra of compound **(a)** in $CDCl_3$ at 25°C, 400MHz.*

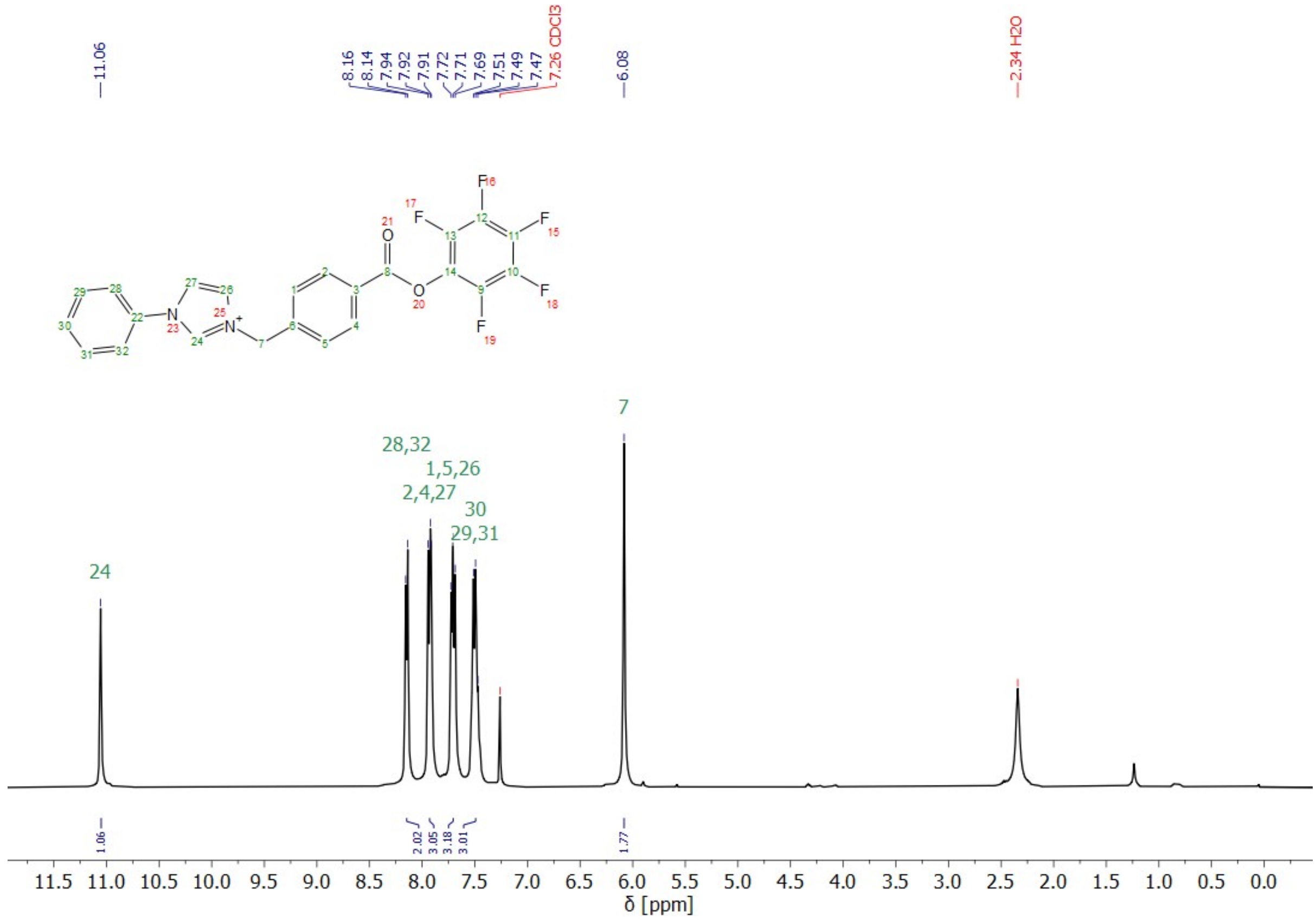

*Figure S4: ^{1}H-NMR spectra of compound **(b)** in $CDCl_3$ at 25°C, 400MHz.*

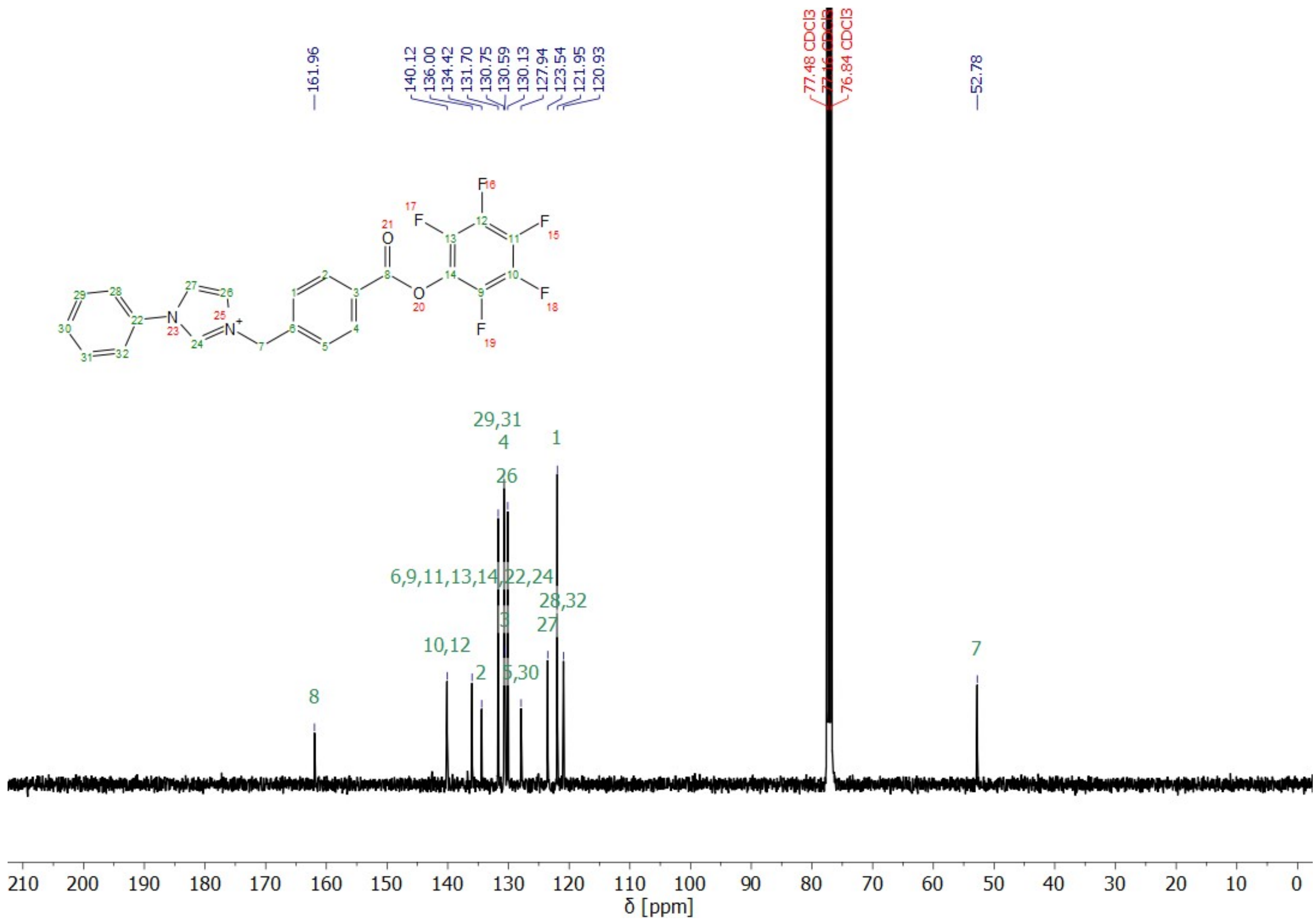

*Figure S5: ^{13}C-NMR spectra of compound **(b)** in $CDCl_3$ at 25°C, 400MHz.*

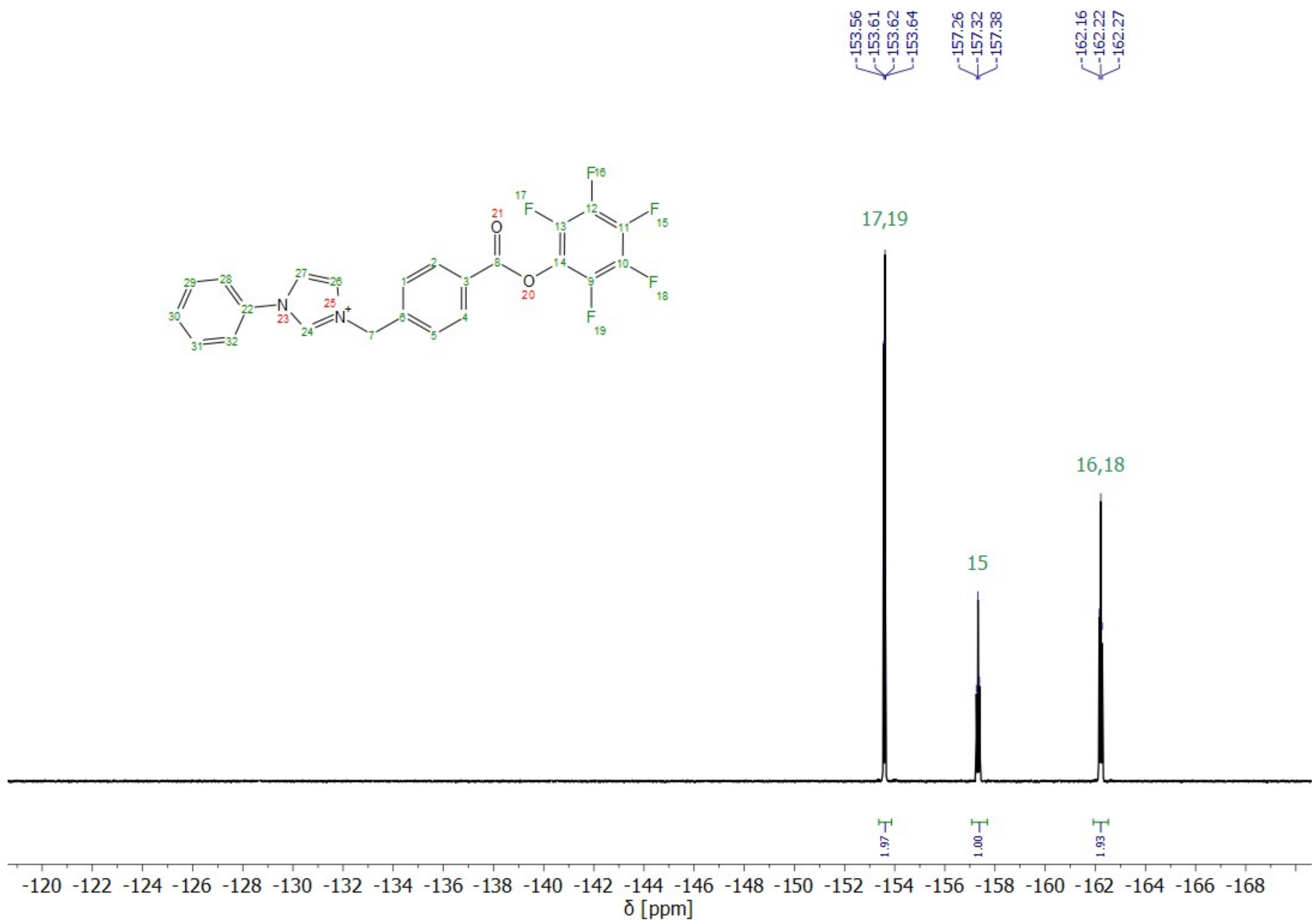

*Figure S6: ^{19}F-NMR spectra of compound **(b)** in $CDCl_3$ at 25°C, 400MHz.*

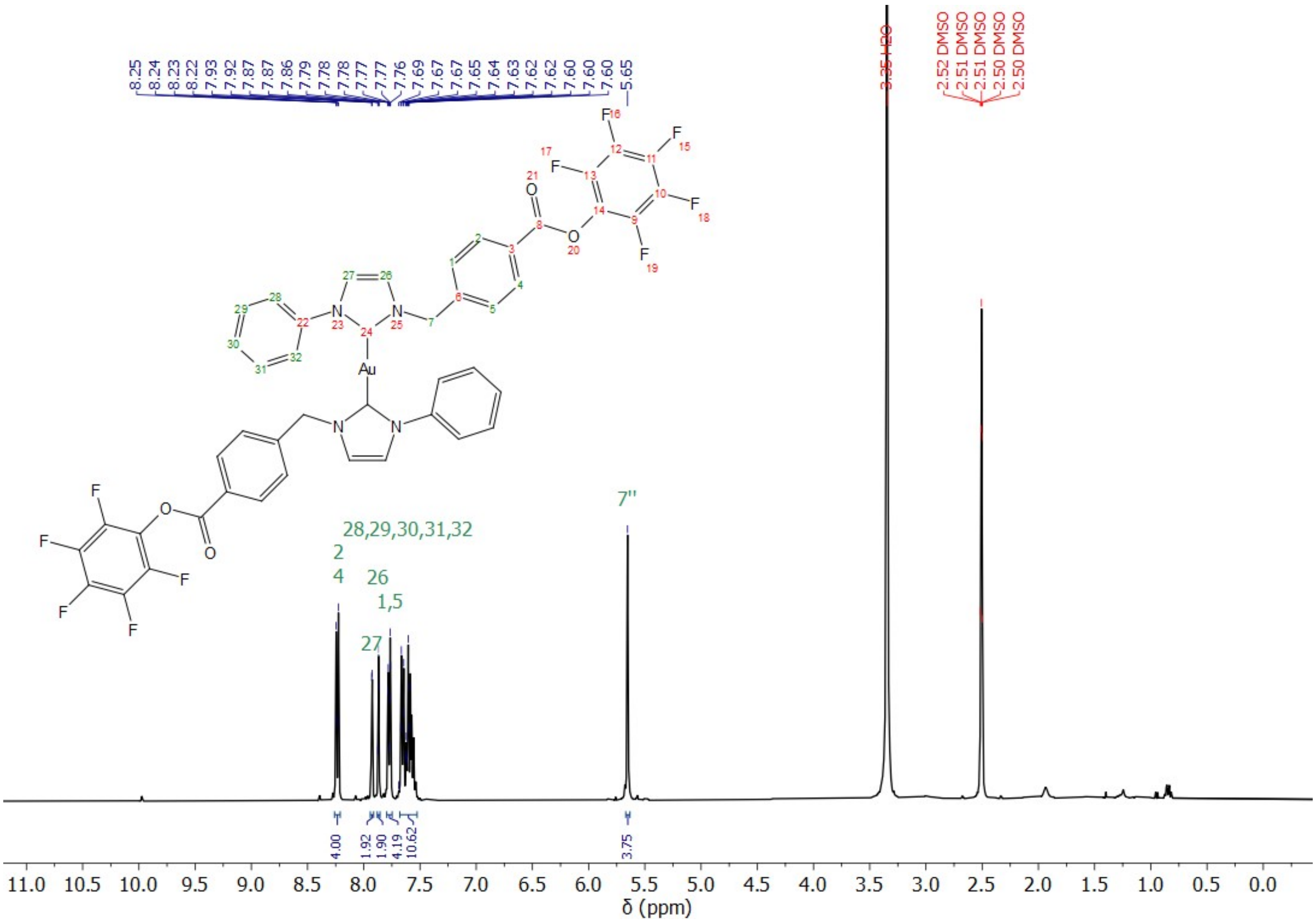

Figure S7: ^{1}H-NMR spectra of compound ***(1)*** *in DMSO-d_6 at 25°C, 400MHz.*

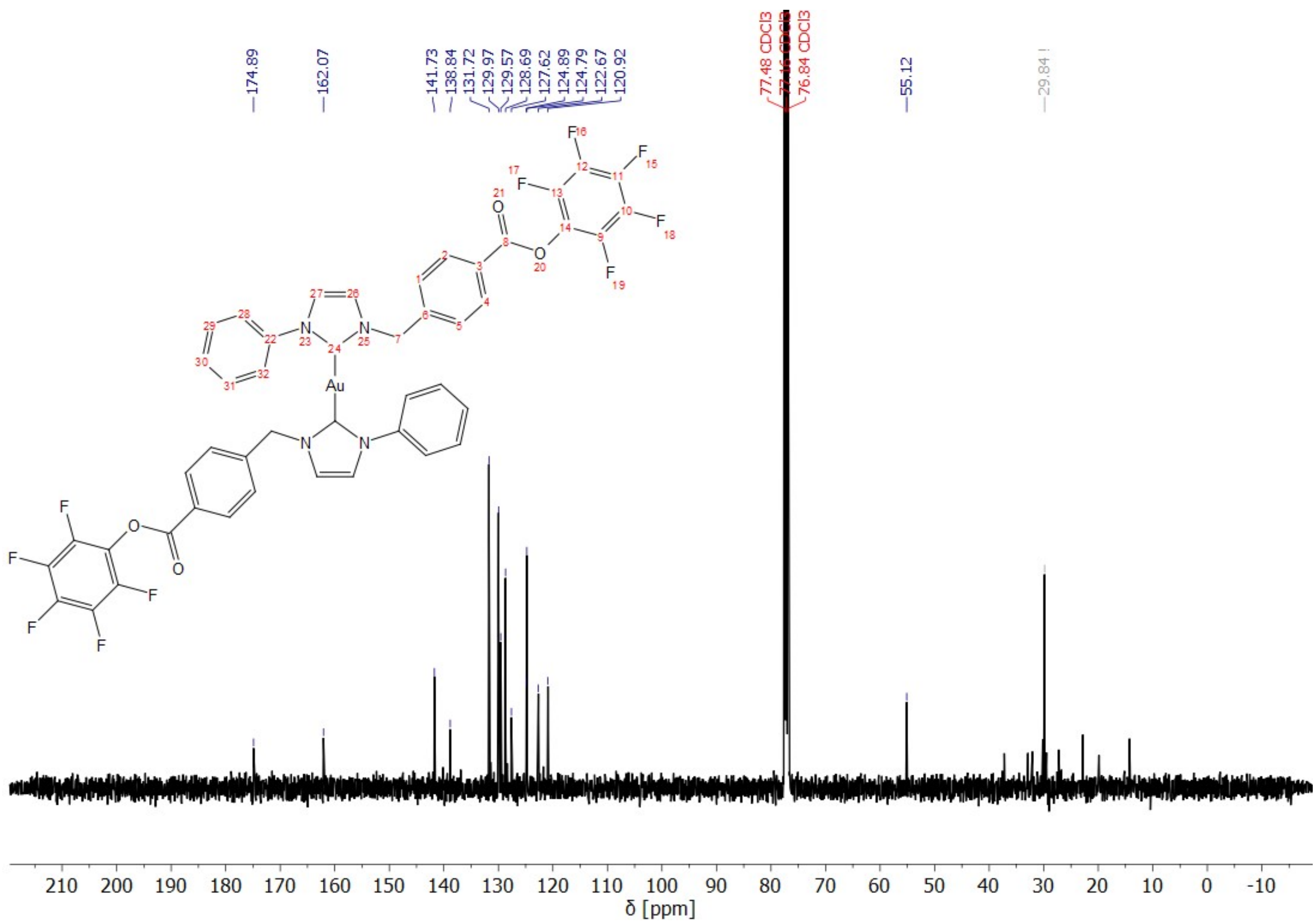

Figure S8: ^{13}C-NMR spectra of compound ***(1)*** *in $CDCl_3$ at 25°C, 400MHz.*

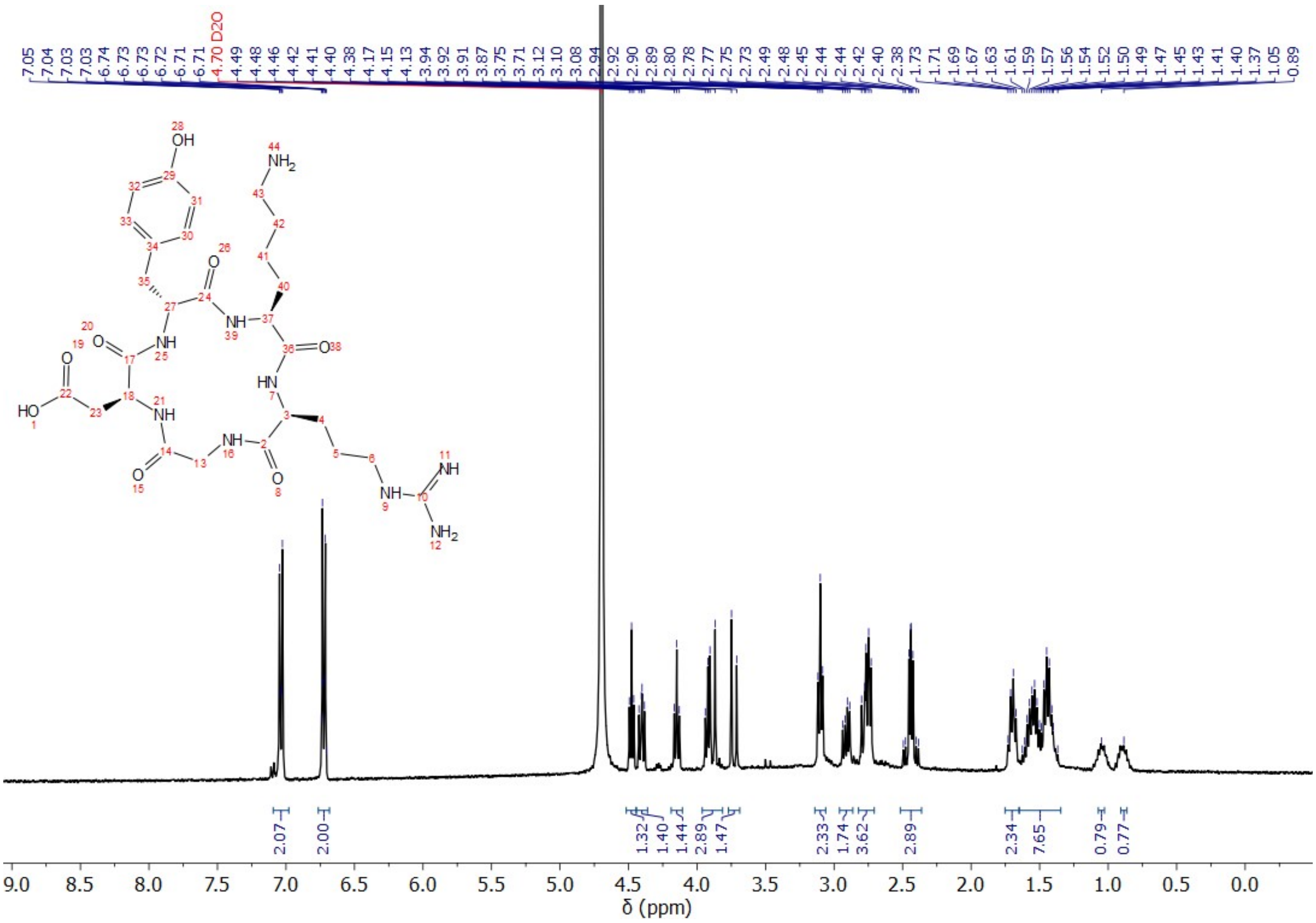

*Figure S9: ^1H-NMR spectra of compound **c(RGDyK)** in D_2O at 25°C, 400MHz.*

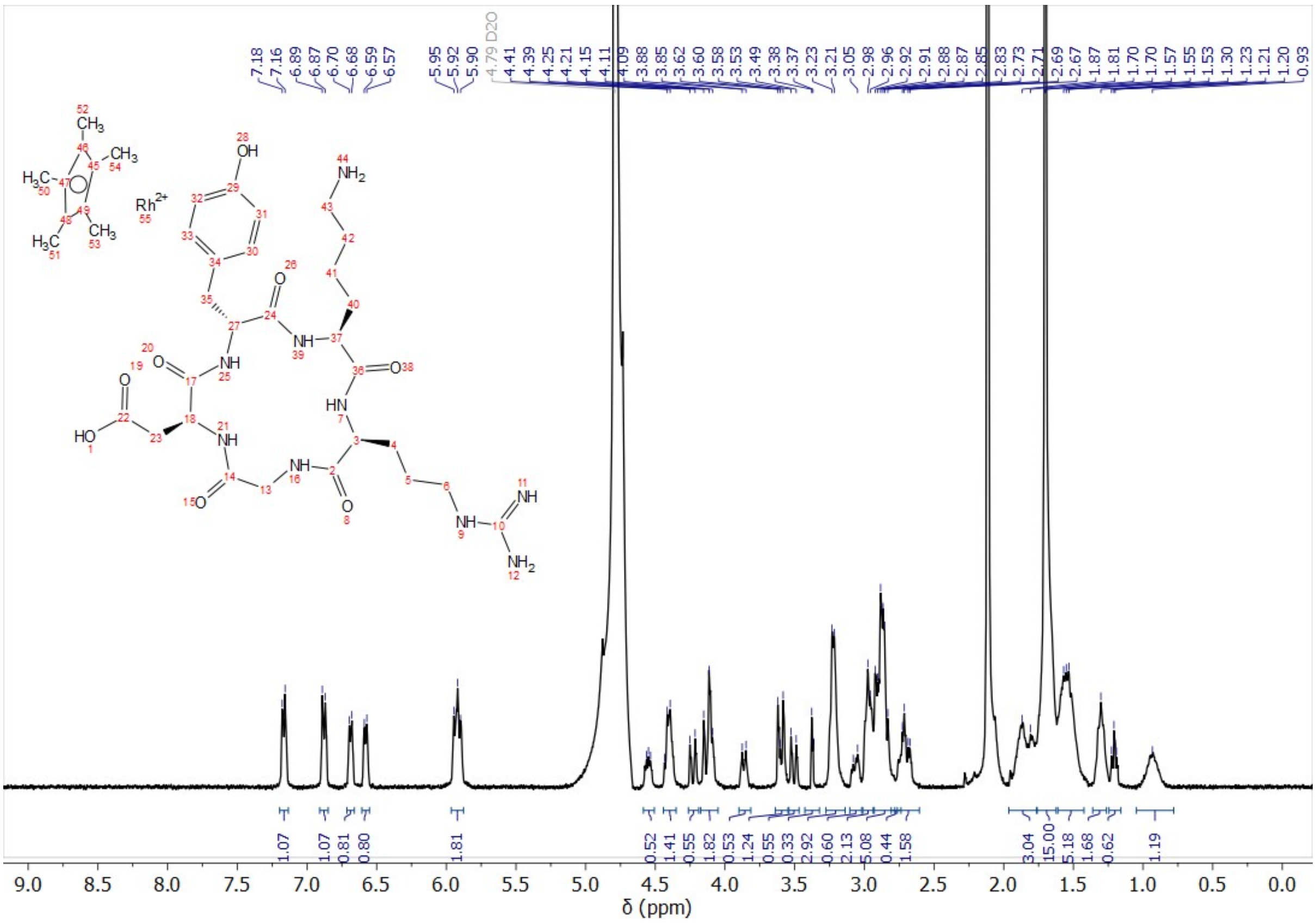

*Figure 10: ^{1}H-NMR spectra of compound **Rh-RGD** in D_2O at 25°C, 400MHz.*

3. HR-MS Spectra

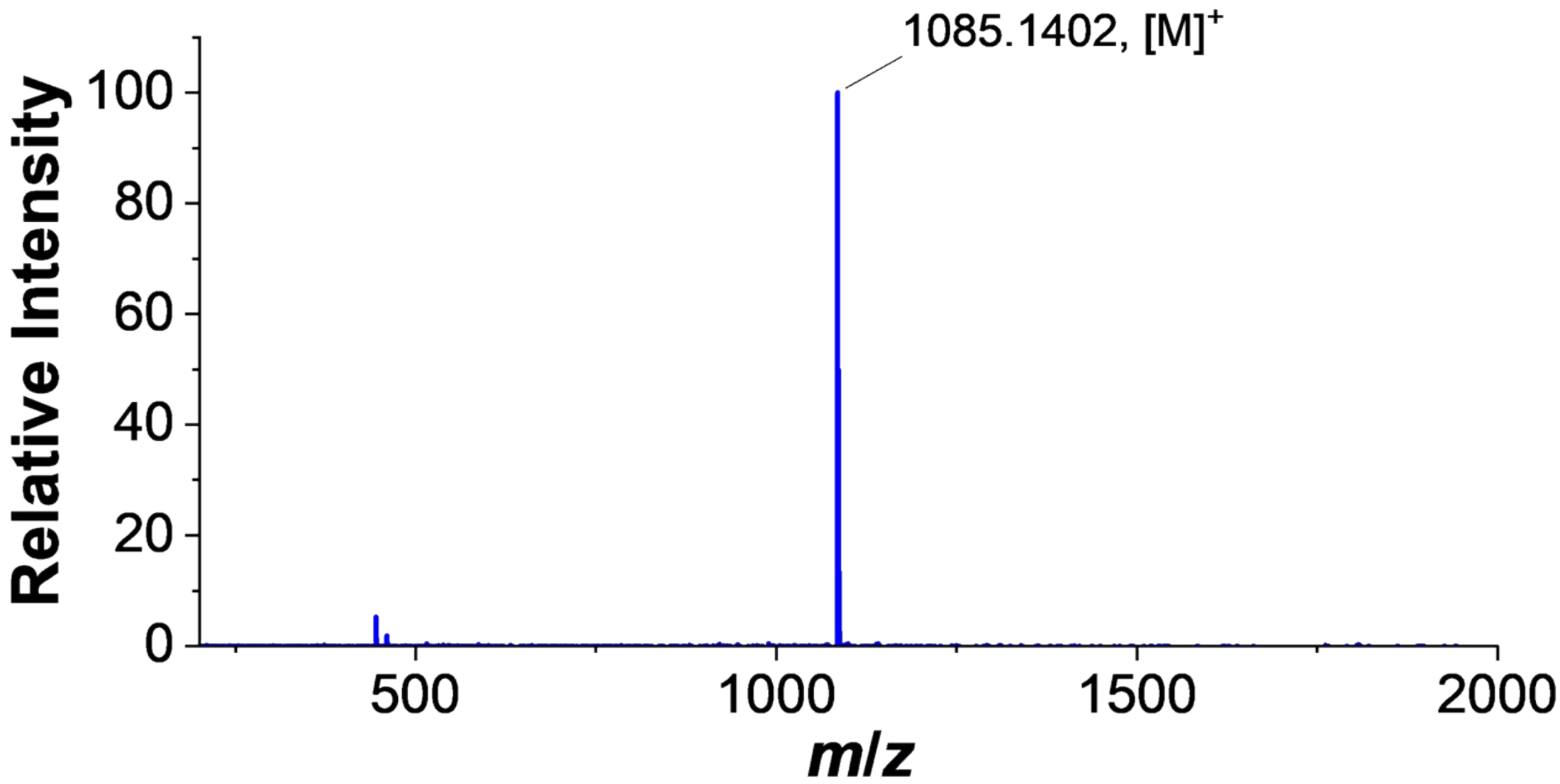

*Figure S11: HR-ESI-MS of **(1)**, m/z calc. for $[C_{46}H_{26}AuF_{10}N_4O_4]^+$: 1085.1460.*

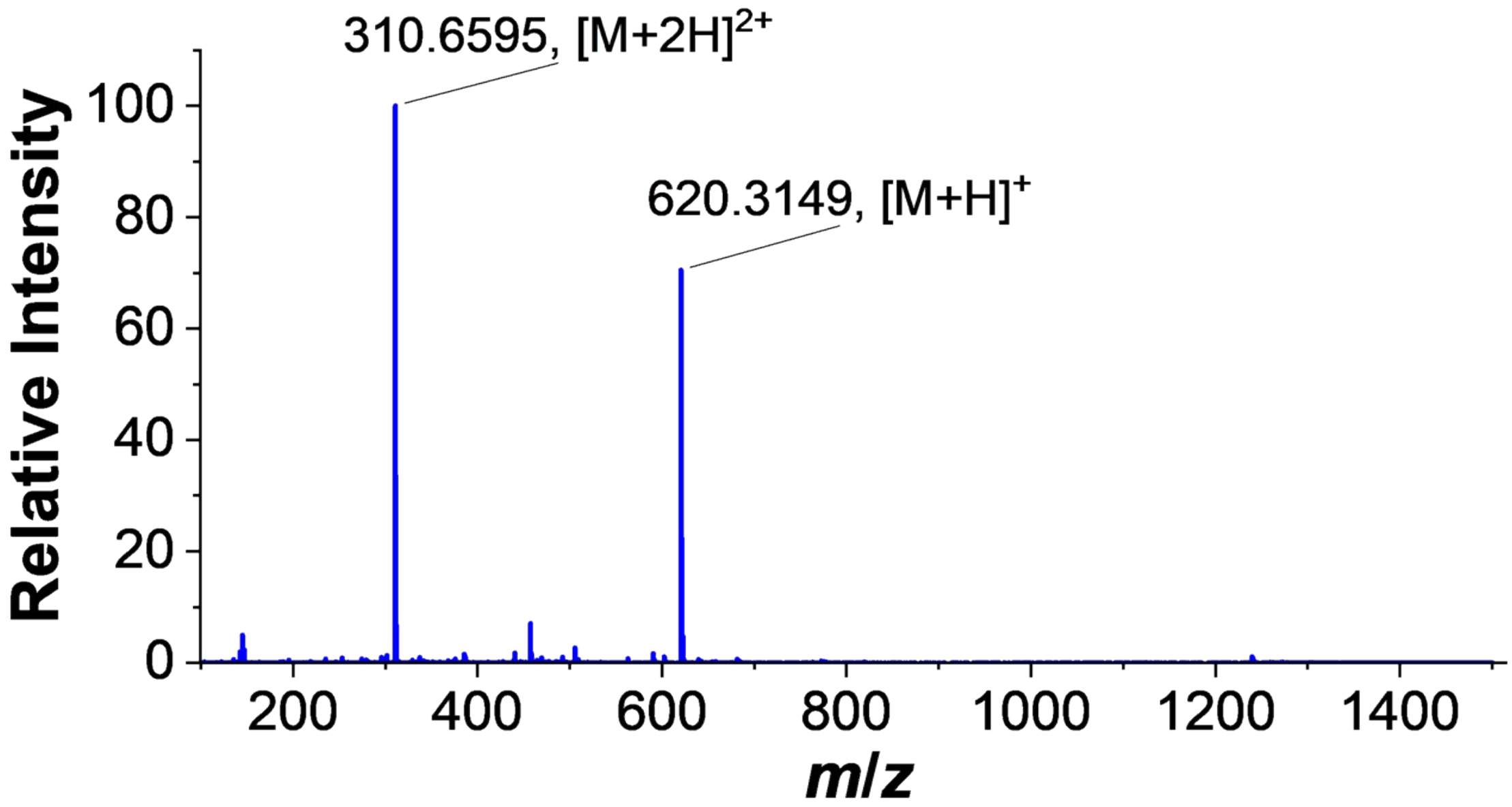

*Figure S12: HR-ESI-MS of **c(RGDyK)**, m/z calc. for $[C_{27}H_{42}N_9O_8]^+$: 620.3151, $[M+H]^+$; $[C_{27}H_{43}N_9O_8]^{2+}$: 310.6612, $[M+2H]^{2+}$.*

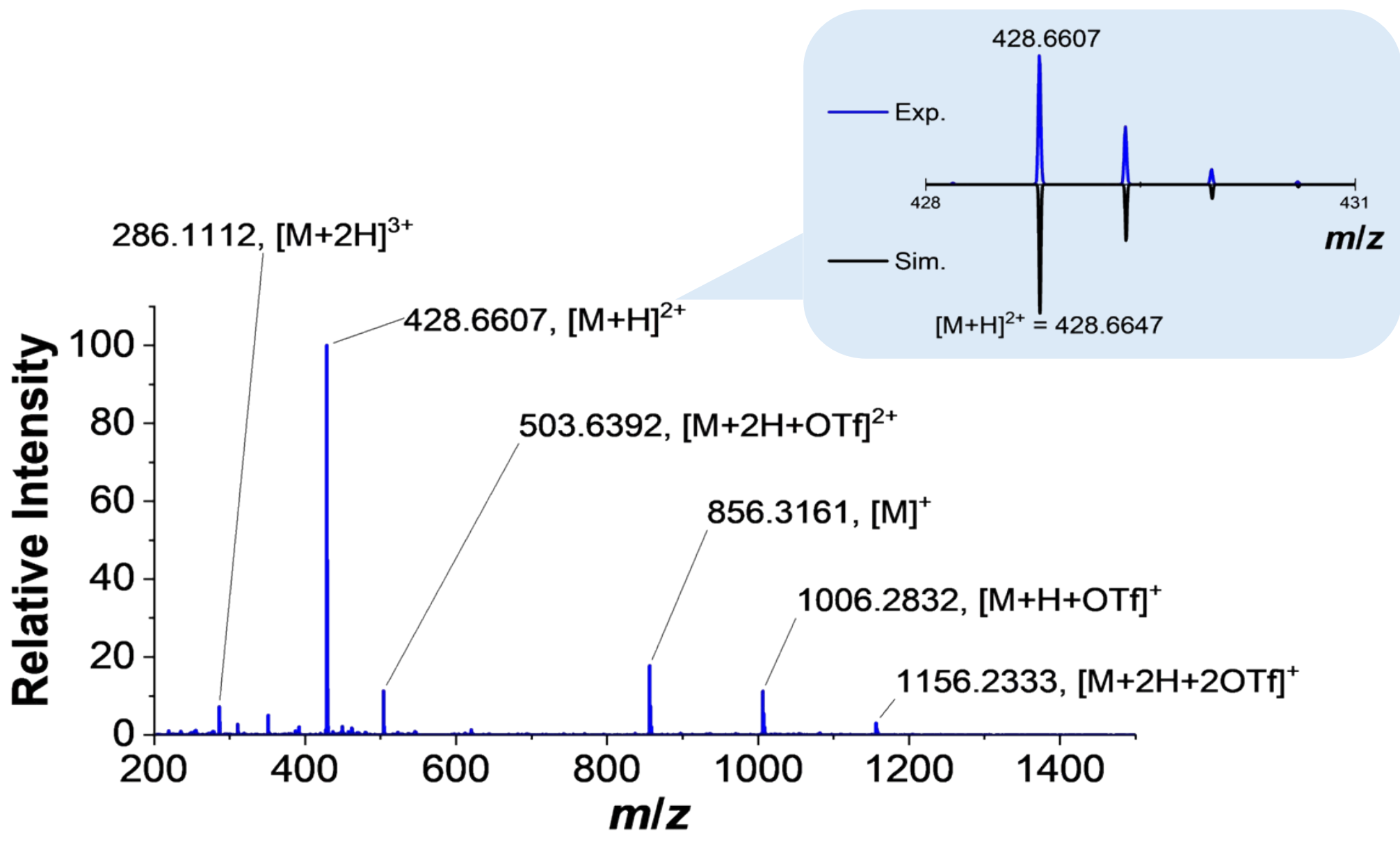

*Figure S13: HR-ESI-MS of **Rh-RGD**; the inset shows the detail of the isotopic pattern of the species m/z calc. for $[C_{37}H_{56}N_9O_8Rh]^{2+}$: 428.6647, $[M+H]^{2+}$.*

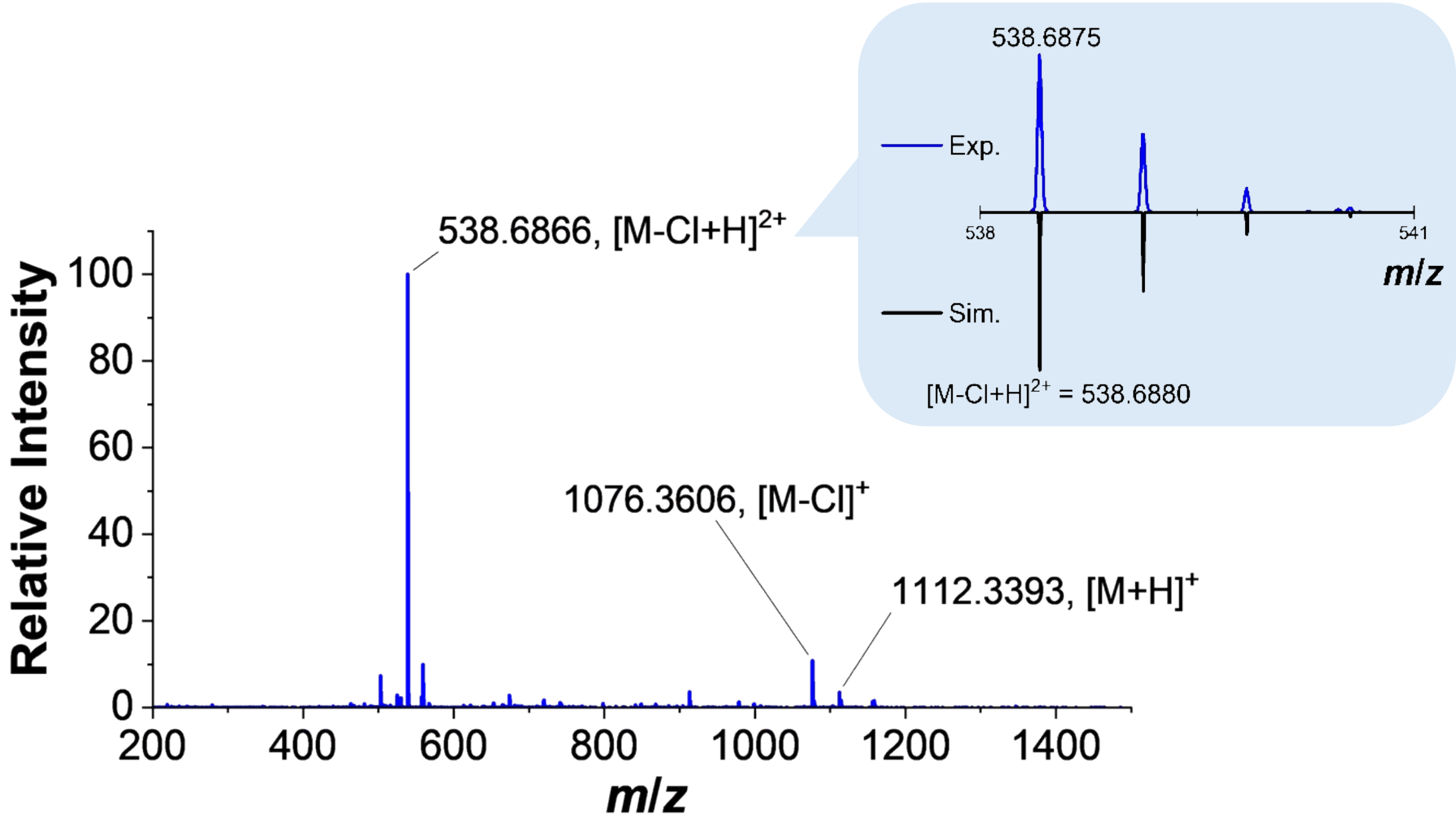

*Figure S14: HR-ESI-MS of **Au-RGD**; the inset shows the detail of the isotopic pattern of the species m/z calc. for $[C_{44}H_{54}AuN_{11}O_9]^{2+}$: 538.6680 $[M+H-Cl]^{2+}$.*

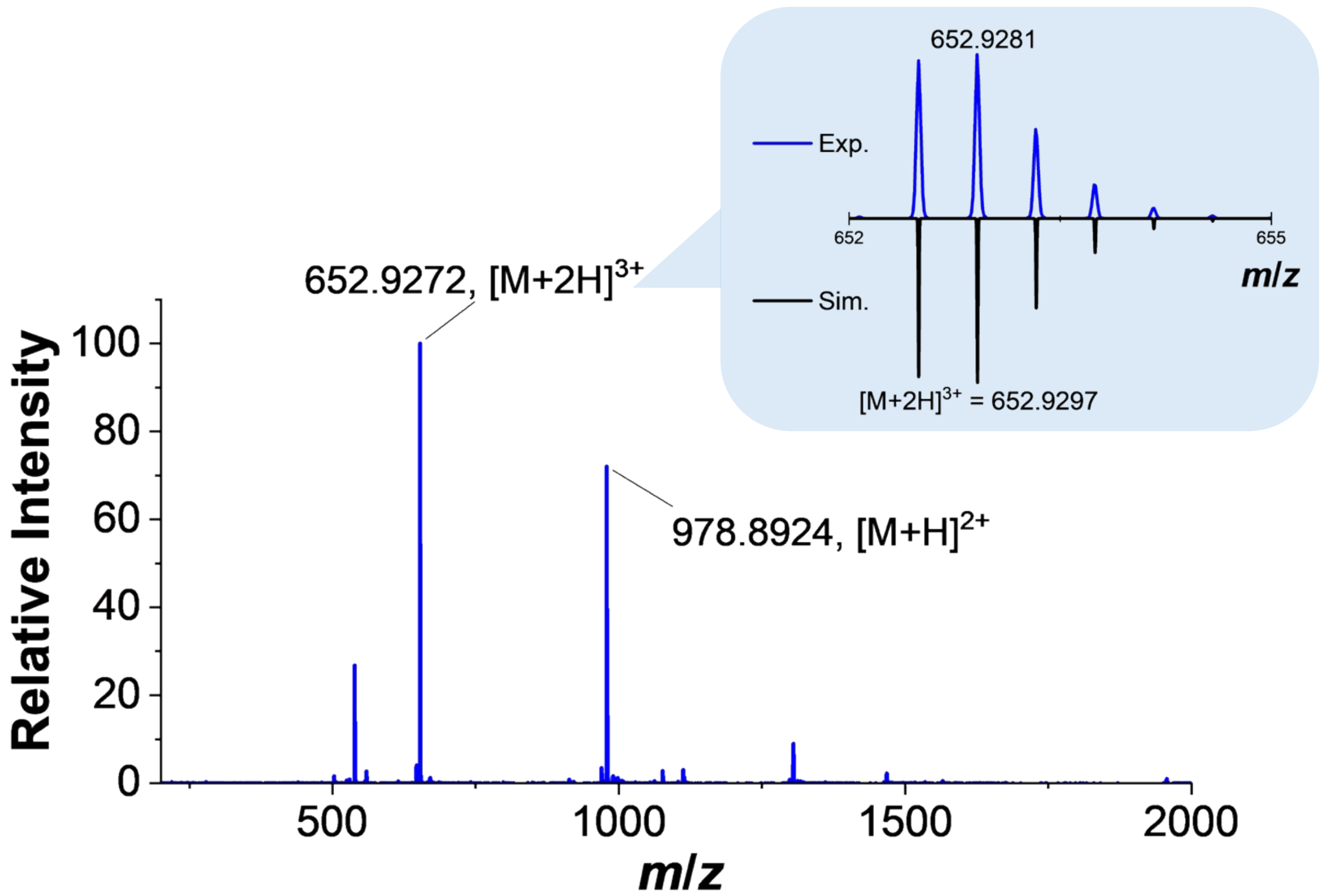

*Figure S15: HR-ESI-MS of **Au-RGD$_2$**; the inset shows the detail of the isotopic pattern of the species m/z calc. for $[C_{88}H_{108}AuN_{22}O_{18}]^{3+}$: 652.5954 $[M+2H]^{3+}$.*

4. Tables

Table 1: Uptake of the OM-conjugates in MDA-MB-231 cells after 24 hours.

Compound	Concentration [µM]	metal /cell protein [nmol/mg]
Au-RGD	10	18.61 ± 3.45
Au-RGD_2	10	0.00 ± 0.00
$[Cp^*Rh(H_2O)_3](OTf)_2$	50	1.89 ± 0.39
Rh-RGD	50	1.08 ± 0.62

5. References

1 R. Uson, A. Laguna, M. Laguna, D. A. Briggs, H. H. Murray and J. P. Fackler Jr., in *Inorganic Syntheses*, John Wiley & Sons, Ltd, 1989, pp. 85–91.
2 M. S. Eisen, A. Haskel, H. Chen, M. M. Olmstead, D. P. Smith, M. F. Maestre and R. H. Fish, *Organometallics*, 1995, **14**, 2806–2812.
3 J. Han, A. F. B. Räder, F. Reichart, B. Aikman, M. N. Wenzel, B. Woods, M. Weinmüller, B. S. Ludwig, S. Stürup, G. M. M. Groothuis, H. P. Permentier, R. Bischoff, H. Kessler, P. Horvatovich and A. Casini, *Bioconjugate Chem.*, 2018, **29**, 3856–3865.
